# Serum neurofilament light chain: a novel biomarker for early diabetic sensorimotor polyneuropathy

**DOI:** 10.1007/s00125-022-05846-8

**Published:** 2022-12-06

**Authors:** Haifa Maalmi, Alexander Strom, Agnese Petrera, Stefanie M. Hauck, Klaus Strassburger, Oliver Kuss, Oana-Patricia Zaharia, Gidon J. Bönhof, Wolfgang Rathmann, Sandra Trenkamp, Volker Burkart, Julia Szendroedi, Dan Ziegler, Michael Roden, Christian Herder, the GDS Group

**Affiliations:** 1 Institute for Clinical Diabetology, German Diabetes Center (Deutsches Diabetes-Zentrum/DDZ), Leibniz Center for Diabetes Research at Heinrich Heine University Düsseldorf, Düsseldorf, Germany; 2 German Center for Diabetes Research (DZD), München-Neuherberg, Germany; 3 Metabolomics and Proteomics Core, Helmholtz Zentrum München, German Research Center for Environmental Health (GmbH), Neuherberg, Germany; 4 Institute for Biometrics and Epidemiology, German Diabetes Center (Deutsches Diabetes-Zentrum/DDZ), Düsseldorf, Germany; 5 Centre for Health and Society, Medical Faculty, Heinrich Heine University, Düsseldorf, Germany; 6 Department of Endocrinology and Diabetology, Medical Faculty and University Hospital Düsseldorf, Heinrich Heine University Düsseldorf, Düsseldorf, Germany; 7 Department of Endocrinology, Diabetology, Metabolism and Clinical Chemistry, Heidelberg University Hospital, Heidelberg, Germany; 8 Institute for Diabetes and Cancer (IDC) & Joint Heidelberg–IDC Translational Diabetes Program, Helmholtz Center Munich, München-Neuherberg, Germany

**Keywords:** Axonal damage, Biomarker, Demyelination, Diabetes, Diabetic neuropathy, Diabetic sensorimotor polyneuropathy, Distal sensorimotor polyneuropathy, Electrophysiological tests, Large fibre, Nerve conduction study, Nerve dysfunction, Nerve injury, Neurofilament light chain, Neurofilaments, Neurological biomarkers, Peripheral nervous system, Peripheral neuropathy, Quantitative sensory tests, Small fibre

## Abstract

**Aims/hypothesis:**

No established blood-based biomarker exists to monitor diabetic sensorimotor polyneuropathy (DSPN) and evaluate treatment response. The neurofilament light chain (NFL), a blood biomarker of neuroaxonal damage in several neurodegenerative diseases, represents a potential biomarker for DSPN. We hypothesised that higher serum NFL levels are associated with prevalent DSPN and nerve dysfunction in individuals recently diagnosed with diabetes.

**Methods:**

This cross-sectional study included 423 adults with type 1 and type 2 diabetes and known diabetes duration of less than 1 year from the prospective observational German Diabetes Study cohort. NFL was measured in serum samples of fasting participants in a multiplex approach using proximity extension assay technology. DSPN was assessed by neurological examination, nerve conduction studies and quantitative sensory testing. Associations of serum NFL with DSPN (defined according to the Toronto Consensus criteria) were estimated using Poisson regression, while multivariable linear and quantile regression models were used to assess associations with nerve function measures. In exploratory analyses, other biomarkers in the multiplex panel were also analysed similarly to NFL.

**Results:**

DSPN was found in 16% of the study sample. Serum NFL levels increased with age. After adjustment for age, sex, waist circumference, height, HbA_1c_, known diabetes duration, diabetes type, cholesterol, eGFR, hypertension, CVD, use of lipid-lowering drugs and use of non-steroidal anti-inflammatory drugs, higher serum NFL levels were associated with DSPN (RR [95% CI] per 1-normalised protein expression increase, 1.92 [1.50, 2.45], *p*<0.0001), slower motor (all *p*<0.0001) and sensory (all *p*≤0.03) nerve conduction velocities, lower sural sensory nerve action potential (*p*=0.0004) and higher thermal detection threshold to warm stimuli (*p*=0.023 and *p*=0.004 for hand and foot, respectively). There was no evidence for associations between other neurological biomarkers and DSPN or nerve function measures.

**Conclusions/interpretation:**

Our findings in individuals recently diagnosed with diabetes provide new evidence associating higher serum NFL levels with DSPN and peripheral nerve dysfunction. The present study advocates NFL as a potential biomarker for DSPN.

**Graphical abstract:**

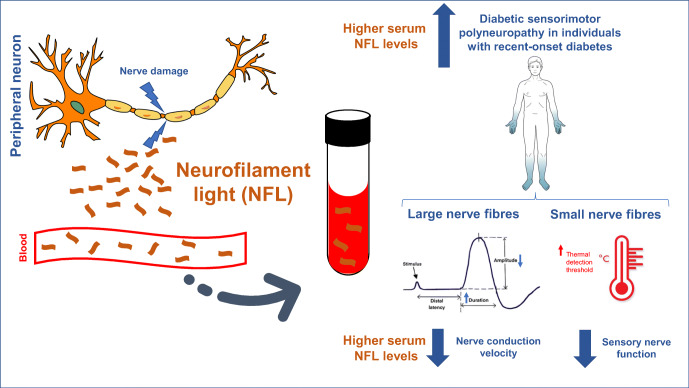

**Supplementary Information:**

The online version of this article (10.1007/s00125-022-05846-8) contains peer-reviewed but unedited supplementary material..

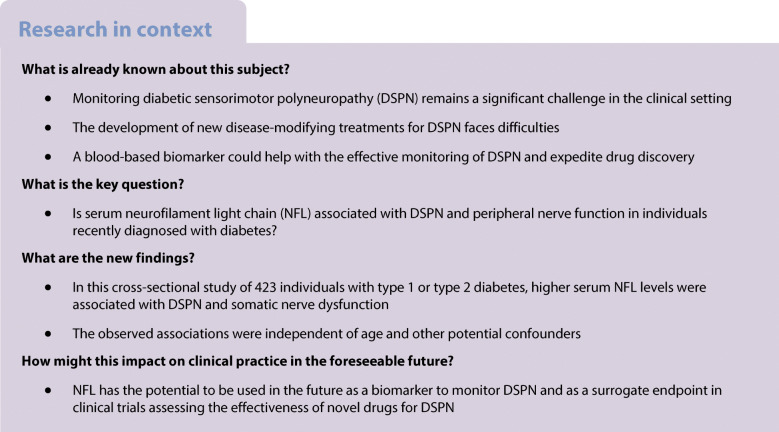



## Introduction

Diabetic sensorimotor polyneuropathy (DSPN) affects individuals with diabetes or impaired glucose tolerance and/or impaired fasting glucose and is characterised by demyelination and axonal loss of peripheral sensory and motor nerves [1]. Despite the significant advances in elucidating the underlying pathogenesis of DSPN, the condition remains underdiagnosed and undertreated in clinical practice and poses major challenges in clinical trials [2, 3]. On the one hand, a delayed diagnosis may foster irreversible neuropathic damage, hamper suitable interventions and increase the risk of associated disabilities and medical costs. On the other hand, the early detection of subclinical or asymptomatic DSPN with electrophysiological or quantitative sensory testing, which could allow for early intervention [4], is time-consuming, expensive, requires expertise and is only accessible in specialised centres. Thus, there is an increased need for simple, easy-to-perform, inexpensive biomarker measurements that can provide clinical information that reasonably reflect early DSPN detected and monitored by peripheral nerve function tests. Beyond difficulties in diagnosis and management, developing new disease-modifying treatments for DSPN is an unmet need, as underlined by numerous failed phase 3 trials [5]. A fundamental reason for these unsuccessful trials is the lack of reproducible and accurate surrogate endpoints predicting the ultimate clinical endpoints such as foot ulceration and amputation [2]. Clinical symptoms and neurological deficits are subjective and, consequently, they have poor reproducibility [6], while electrophysiological studies represent US Food and Drug Administration (FDA)-approved objective surrogate endpoints [7], albeit their reproducibility has been questioned because of high inter-observer variability [6].

Therefore, there is a pressing demand to identify and validate novel biomarkers to monitor DSPN and facilitate drug discovery. Ideally, these biomarkers should be blood-based and easy to measure to expedite their implementation in the clinical setting. Additionally, their measurement should be objective, reproducible and accurate to allow their use as biomarkers for the progression of DSPN in clinical trials.

The neurofilament light chain (NFL) is a cytoskeletal component of mature neurons that provides structural stability and determines axonal diameter [8, 9]. NFL is more abundant in large myelinated axons facilitating a faster conduction velocity. Upon axonal injury, NFL is released from axons into the circulation [10]. The efficacy of serum NFL as a biomarker of neuroaxonal damage emerged initially in multiple sclerosis, where it serves for prognosis and treatment monitoring [10, 11]. Currently, there is growing scientific evidence associating serum NFL with other neurodegenerative diseases [12] and peripheral neuropathies in humans [13–25] and animals [26–28]. However, evidence of a potential association between NFL and DSPN is scarce. One recent preliminary study indicated inverse correlations between serum NFL and some nerve function measures in individuals with type 2 diabetes who had a known diabetes duration of 3 years or less [29]. An earlier study reported higher blood *NFL* (also known as *NEFL*) mRNA levels in people with impaired glucose tolerance and/or impaired fasting glucose and peripheral neuropathy than those without [30]. However, whether serum NFL is associated with prevalent DSPN in individuals with type 1 or type 2 diabetes of a very short known duration remains unknown.

Therefore, the primary aim of the present study was to investigate associations of serum NFL with prevalent DSPN and peripheral nerve function in recently diagnosed type 1 and type 2 diabetes. We hypothesised that higher serum NFL levels are associated with prevalent DSPN and nerve dysfunction. Given that we measured serum NFL with proximity extension assay technology in a 92-biomarker panel, the secondary aim of our study was to explore potential associations of other biomarkers in this panel with DSPN.

## Methods

### Study design and study population

The German Diabetes Study (GDS) is an ongoing observational prospective study that evaluates the natural course of recently diagnosed diabetes and explores prognostic factors and mechanisms leading to diabetes-related complications [31]. Individuals aged 18–69 years at the baseline examination with a known diabetes duration of less than 1 year are eligible to participate, while individuals with overt neurological diseases, such as multiple sclerosis, dementia or psychiatric disorders, are excluded. Diabetes is diagnosed according to the ADA criteria [32]. At baseline, all participants undergo a comprehensive examination consisting of clinical tests, a face-to-face interview, standardised written questionnaires and detailed laboratory measurements.

The GDS is conducted according to the Declaration of Helsinki, approved by the ethics committee of Heinrich Heine University, Düsseldorf, Germany (ref. 4508) and was registered with ClinicalTrials.gov (registration no. NCT01055093). All participants provided written informed consent.

This cross-sectional analysis focused on 503 participants recruited consecutively from 2005 to 2011. From these 503 participants, we excluded 51 participants with missing data on one of the neurological variables used to define DSPN, two participants with diabetes forms other than type 1 or type 2 diabetes and 36 participants with missing covariates, leaving 423 participants with complete data for analysis (ESM Fig. 1).

### Measurement of serum NFL

NFL was measured in serum of fasting participants at baseline using the Olink Target 96 Neuro Exploratory multiplex assay panel (Olink Proteomics, Uppsala, Sweden; NFL Uniprot ID: P07196 and NFL Olink ID: OID05206) based on proximity extension assay technology. The relative concentration of serum NFL is expressed as normalised protein expression (NPX) values, which are comparable in their distribution to log_2_-transformed protein concentrations. A detailed description of all 92 biomarkers in the panel is given in ESM Table 1; data cleaning steps that left 60 biomarkers, in addition to NFL, for exploratory analysis are described in ESM Fig. 2. We first excluded 29 biomarkers because of missing data for ≥25%. Then, we further excluded two biomarkers because of inter-assay CV >25%.

### Assessment of peripheral neuropathy

All participants underwent nerve conduction studies (NCSs) and quantitative sensory testing (QST) as previously described [33, 34]. Motor nerve conduction velocity (MNCV) was measured in the peroneal, median and ulnar nerves, while sensory nerve conduction velocity (SNCV) and sensory nerve action potential (SNAP) were measured in the sural, median and ulnar nerves. All nerve stimulations were performed using surface electrodes (Nicolet VikingQuest; Natus Medical, San Carlos, CA, USA) after warming up feet and lower legs to ensure that skin temperature was 33–34°C. QST was evaluated by thermal detection thresholds (TDTs) to warm and cold stimuli at the thenar eminence and dorsum of the foot using the method of limits (TSA-II NeuroSensory Analyzer; Medoc, Ramat Yishai, Israel). Neurological examination was performed using the Neuropathy Disability Score (NDS) for neuropathic signs and the Neuropathy Symptom Score (NSS) for neuropathic symptoms [35]. Stages of DSPN were defined, according to the Toronto Consensus criteria [36], as subclinical, confirmed asymptomatic and confirmed symptomatic as previously described [33].

### Covariates

Body weight (kg), waist circumference (cm) and height (m) were measured at enrolment. Information on known diabetes duration (days), presence of chronic diseases, use of non-steroidal anti-inflammatory drugs (NSAIDs) (yes/no) and lipid-lowering drugs (yes/no) was obtained during a face-to-face interview. Hypertension was defined as either systolic BP ≥140 mmHg, diastolic BP ≥90 mmHg or use of antihypertensive medication. Self-reported myocardial infarction, peripheral arterial occlusive disease, cerebrovascular disease or stroke was used to define the presence of CVD. Total cholesterol and HbA_1c_ were measured according to standardised laboratory procedures in blood samples collected at baseline after overnight fasting [31]. Diabetes-related autoantibodies were measured for each participant. The eGFR was calculated from serum creatinine and cystatin C using the Chronic Kidney Disease Epidemiology (CKD-EPI) equation [37].

### Statistical analyses

Data are presented as median (25th, 75th percentiles), mean ± SD, or percentages in descriptive statistics. Differences in characteristics according to DSPN status were tested with generalised linear regression analyses allowing for different group variances using the SAS procedure GLIMMIX and with the χ^2^ test, while differences in serum NFL levels between the two groups were tested with ANCOVA to allow adjustment for age at diagnosis. In addition, differences in means and 95% CIs were also calculated. Spearman’s rank correlation tests estimated the non-adjusted and age-adjusted correlations of serum NFL with demographic and metabolic variables.

In primary analyses, we assessed the associations of serum NFL with DSPN (primary endpoint) and with nerve function measures (exploratory secondary endpoints). First, the association of serum NFL (independent variable, by 1-NPX increase, by 1-SD increase and by tertiles) with DSPN (binary dependent variable) was assessed using Poisson regression models with a robust error variance. Model 1 was adjusted for sex and age at diagnosis. Model 2 was additionally adjusted for waist circumference, height, HbA_1c_, known diabetes duration, diabetes type, eGFR, total cholesterol, hypertension, CVD, lipid-lowering drugs and NSAIDs. Based on our previous studies, these covariates were defined a priori as covariates that may affect nerve conduction [38, 39]. Associations were estimated with RRs of DSPN and their corresponding 95% CIs. Receiver operating characteristic (ROC) curves were used to assess the predictive performance of serum NFL. We conducted the following sensitivity analyses to test the robustness of our results: (1) we substituted waist circumference and height with BMI; (2) we excluded individuals with type 1 diabetes; and (3) we excluded participants with prevalent self-reported CVD (*n*=10). In addition, we tested for interaction with diabetes type. Second, the associations of serum NFL with MNCVs and SNCVs were assessed using multivariable linear regression models, while associations with SNAPs and TDTs were assessed using quantile regression models, which do not make assumptions about the residual distribution. These analyses were adjusted for the same covariates as in the Poisson regression models, and associations were estimated with β estimates and their corresponding 95% CIs. In addition, for each participant, individual MNCVs, SNCVs and SNAPs were standardised and summarised in sum scores, which were used as additional secondary outcomes analysed using multivariable linear regression models. *Z* scores were calculated by subtracting the mean value of nerve conduction velocity (NCV) in the study population from the value in the individual and dividing the result by the SD. We constructed an ‘MNCV sum score’ based on MNCVs, an ‘SNCV sum score’ based on SNCVs, and a ‘total NCV sum score’ based on MNCVs, SNCVs and SNAPs. The sum scores combine information about the NCVs of different nerves by giving equal weight to each nerve, allowing for a more comprehensive assessment of nerve function in an individual [39, 40]. Primary analyses addressing a pre-planned hypothesis on NFL with primary and secondary exploratory endpoints were not adjusted for multiple testing.

In a secondary hypothesis-free exploratory analysis, all analyses mentioned above for NFL were performed for the remaining 60 biomarkers of the Olink Target 96 Neuro Exploratory multiplex assay. These hypothesis-free analyses were adjusted for multiple testing with the Bonferroni method (a recommended approach when many tests are carried out without pre-planned hypotheses) [41] and a Bonferroni-corrected *p*<0.0008 (0.05/61) indicated significant associations.

All statistical analyses were carried out with SAS version 9.4 (SAS Institute, Cary, NC, USA), and *p* values <0.05 were considered indicators of a statistically significant correlation, difference or association unless otherwise stated. The visualisation was carried out with RStudio version 4.0.5 (https://posit.co/download/rstudio-desktop).

## Results

### Participants’ characteristics

Participants with different DSPN severity stages (subclinical [*n*=41], confirmed asymptomatic [*n*=11] and confirmed symptomatic [*n*=14]) were merged to create an overall DSPN group (*n*=66, 16%), which was compared with participants without DSPN (*n*=357, 84%).

**Fig. 1:**
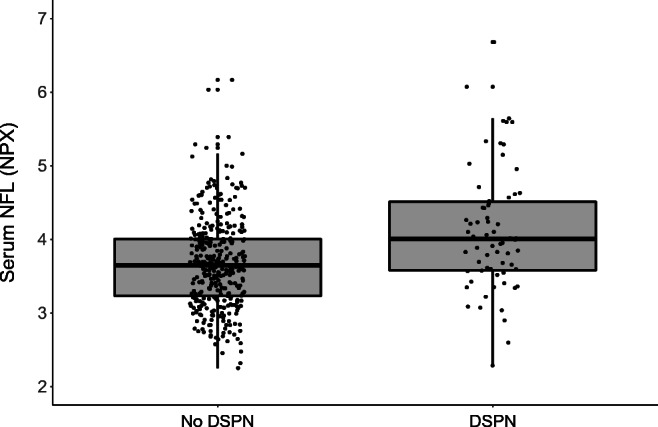
Serum NFL according to DSPN status. The boxplots with jittered data points show the distribution of serum NFL according to DSPN status. The line that divides the box into two parts represents the median of the data. The top and bottom of the box show the upper (Q3) and lower (Q1) quartiles. The extreme line shows Q3+1.5×IQR to Q1−1.5×IQR. Serum NFL is expressed as NPX values

Table 1 shows the demographic and clinical characteristics of the study population overall (*n*=423) and stratified by DSPN status. Participants with DSPN were more likely to be men, to be older, and to have higher waist circumference and height than individuals without DSPN. They were also more likely to have type 2 diabetes, a shorter known diabetes duration and use lipid-lowering drugs. However, there was no evidence for differences in HbA_1c_, cholesterol levels or proportion of individuals with hypertension, CVD and use of NSAIDs between those with and without DSPN. Serum NFL levels (median [25th percentile, 75th percentile]) were higher in participants with DSPN (4.0 [3.6, 4.5]) compared with those without DSPN (3.7 [3.2, 4.0], *p*<0.0001) (Table 1 and Fig. 1), and higher in each DSPN stage compared with the group without DSPN despite the small sample size (ESM Fig. 3).

**Table 1:** Clinical characteristics in the total study sample and stratified by DSPN

Characteristic	Total sample (*n*=423)	DSPN			
		Present (*n*=66, 16%)	Absent (*n*=357, 84%)	*p* value	Effect size^a^
Age, years	46.1 ± 14.4	49.1 ± 12.5	45.6 ± 14.7	0.043	3.5 (0.1, 6.9)
Sex (men/women), %	65/35	85/15	61/39	0.0002	0.3 (0.1, 0.6)
BMI, kg/m^2^	28.9 ± 6.2	30.2 ± 6.0	28.7 ± 6.2	0.060	1.5 (−0.1, 3.1)
Waist circumference, cm	98.0 ± 16.6	103.2 ± 16.1	97.1 ± 16.5	0.005	6.1 (1.8, 10.4)
Height, cm	173.7 ± 9.7	176.1 ± 9.3	173.3 ± 9.7	0.029	2.7 (0.3, 5.2)
Diabetes type (type 1/type 2), %	37/63	20/80	40/60	0.002	2.7 (1.4, 5.2)
Diabetes duration, days	195.8 ± 94.1	167.0 ± 84.5	201.1 ± 94.9	0.003	−34.1 (−56.8, −11.4)
HbA_1c_, mmol/mol	48.0 ± 12.3	50.2 ± 14.4	47.6 ± 11.8	0.165	2.6 (−1.1, 6.3)
HbA_1c_, %	6.5 ± 1.1	6.7 ± 1.3	6.5 ± 1.1	0.165	0.2 (−0.1, 0.6)
eGFR, ml/min per 1.73 m^2^	94.7 ± 16.4	93.5 ± 16.7	94.9 ± 16.4	0.527	−1.4 (−2.9, 5.8)
Total cholesterol, mmol/l	196.2 ± 42.7	191.7 ± 41.6	197.1 ± 42.9	0.333	−5.4 (−16.4, 5.6)
Triacylglycerols, mmol/l	1.2 (0.8, 1.9)	1.2 (0.8, 1.7)	1.1 (0.7, 1.9)	0.686	1.0 (0.9, 1.2)
Hypertension, %	57	67	55	0.083	0.6 (0.3, 1.0)
CVD, %	5	6	5	0.762	0.8 (0.3, 2.5)
Glucose-lowering drugs, %^b^				0.045	1.9 (1.0, 3.8)
None	28	18	30		
Metformin	28	45	24		
Insulin	36	21	39		
Other	8	15	6		
Lipid-lowering drugs, %	13	23	12	0.017	0.4 (0.2, 0.9)
NSAIDs, %	13	12	13	0.913	1.0 (0.5, 2.3)
NFL, NPX^c^	3.7 (0.7)	4.1 (0.8)	3.7 (0.6)	<0.0001	0.4 (0.2, 0.7)

Data are presented as median (25th, 75th percentiles), mean ± SD, or percentages

^a^Effect sizes of continuous variables refer to the difference of mean values between the groups with present and absent DSPN and their 95% confidence intervals; effect sizes of categorical variables refer to the odds ratios and their corresponding 95% CIs

^b^None vs metformin, insulin and other

^c^The difference between present/absent DSPN groups was tested with an ANCOVA adjusted for age

*p* values correspond to comparisons of present vs absent DSPN

Serum NFL levels were positively correlated with age at diagnosis (*r*=0.61, *p*<0.0001) (ESM Fig. 4) but correlations with BMI, waist circumference, height, HbA_1c_, total cholesterol and eGFR varied before and after adjustment for age (ESM Table 2).

### Association of serum NFL with DSPN

Table 2 shows the association between serum NFL and prevalent DSPN. In model 1, adjusted for sex and age at diagnosis, higher serum NFL levels were positively associated with prevalent DSPN (RR [95% CI] per 1-NPX increase, 1.94 [1.51, 2.49]; *p*<0.0001). This positive association remained constant in model 2 (the fully adjusted model) (RR [95% CI] 1.92 [1.50, 2.45]; *p*<0.0001). Similarly, participants in the second and third tertiles of serum NFL showed higher adjusted RRs for prevalent DSPN compared with those in the lowest tertile (RR [95% CI] 2.63 [1.31, 5.29] for tertile 2 and 4.28 [1.50, 12.17] for tertile 3; *p*_trend_=0.007). ESM Fig. 5 shows that the AUC of serum NFL for DSPN as outcome was 0.66 (95% CI 0.59, 0.74; *p*<0.0001).

**Table 2:** RRs and 95% CIs for the association of serum NFL with DSPN

Model	Serum NFL (continuous)				Tertiles of serum NFL			
	1-NPX increase	*p* value	1-SD increase	*p* value	T1	T2	T3	*p*_*trend*_
Model 1^a^	1.94 (1.51, 2.49)	<0.0001	1.55 (1.32, 1.83)	<0.0001	Ref.	2.57 (1.23, 5.36)	4.13 (1.37, 12.42)	0.012
Model 2^b^	1.92 (1.50, 2.45)	<0.0001	1.54 (1.31, 1.81)	<0.0001	Ref.	2.63 (1.31, 5.29)	4.28 (1.50, 12.17)	0.007

^a^Model 1: adjusted for sex and age at diagnosis

^b^Model 2: additionally adjusted for waist circumference, height, HbA_1c_, known diabetes duration, diabetes type, eGFR, total cholesterol, hypertension, CVD, lipid-lowering drugs and NSAIDs

### Sensitivity analyses

The association of serum NFL levels with prevalent DSPN remained consistent when waist circumference and height were substituted with BMI (RR [95% CI] per 1-NPX increase 1.88 [1.47, 2.40]) and when the analyses were restricted to participants with type 2 diabetes (RR [95% CI] 1.96 [1.51, 2.54]). These associations remained robust when the analysis was repeated after excluding participants with prevalent CVD (RR [95% CI] 1.87 [1.46, 2.39]). Interaction by diabetes type was not significant (*p*=0.99).

### Associations of serum NFL with peripheral nerve function tests

Table 3 displays the associations of serum NFL with nerve conduction measures. Higher serum NFL levels were associated with slower MNCV (all *p*<0.0001) and SNCV (all *p*≤0.03) in all nerves and lower NCV sum scores (all *p*<0.0001). These associations were observed in model 1 and remained consistent in model 2. The highest estimates were found for peroneal MNCV and sural SNCV. In addition, higher serum NFL levels were associated with lower sural SNAP (*p*=0.0004) only in model 2. Table 4 displays the associations of serum NFL with TDT. Higher serum NFL levels were only associated with higher TDT to warm stimuli on the hand and foot in model 2 (*p*=0.023 and *p*=0.004 for hand and foot, respectively).

**Table 3:** Associations of serum NFL with nerve conduction measures

Outcome	Model 1^a^				Model 2^b^			
	Per 1-NPX		Per 1-SD		Per 1-NPX		Per 1-SD	
	β (95% CI)	*p* value	β (95% CI)	*p* value	β (95% CI)	*p* value	β (95% CI)	*p* value
Median MNCV	−1.31 (−1.99, −0.62)	0.0002	−0.86 (−1.32, −0.41)	0.0002	−1.36 (−2.04, −0.68)	<0.0001	−0.90 (−1.35, −0.45)	<0.0001
Ulnar MNCV	−1.83 (−2.70, −0.95)	<0.0001	−1.21 (−1.79, −0.63)	<0.0001	−1.82 (−2.71, −0.93)	<0.0001	−1.20 (−1.80, −0.61)	<0.0001
Peroneal MNCV	−2.32 (−3.04, −1.59)	<0.0001	−1.54 (−2.02, −1.05)	<0.0001	−2.54 (−3.23, −1.84)	<0.0001	−1.69 (−2.15, −1.22)	<0.0001
Median SNCV	−1.26 (−2.37, −0.14)	0.030	−0.83 (−1.57, −0.09)	0.030	−1.30 (−2.44, −0.16)	0.030	−0.86 (−1.62, −0.10)	0.030
Ulnar SNCV	−1.53 (−2.40, −0.65)	0.0007	−1.01 (−1.59, −0.43)	0.0007	−1.37 (−2.25, −0.48)	0.003	−0.90 (−1.50, −0.31)	0.003
Sural SNCV	−1.79 (−2.68, −0.91)	<0.0001	−1.19 (−1.78, −0.60)	<0.0001	−2.04 (−2.92, −1.16)	<0.0001	−1.35 (−1.94, −0.77)	<0.0001
Median SNAP	−0.75 (−1.37, −0.14)	0.016	−0.50 (−0.91, −0.09)	0.016	−0.45 (−0.95, 0.04)	0.075	−0.30 (−0.63, 0.03)	0.075
Ulnar SNAP	−0.75 (−1.34, −0.16)	0.012	−0.50 (−0.89, −0.10)	0.012	−0.44 (−1.07, 0.18)	0.168	−0.29 (−0.71, 0.12)	0.168
Sural SNAP	−0.99 (−2.06, 0.08)	0.070	−0.65 (−1.37, 0.05)	0.070	−1.58 (−2.45, −0.71)	0.0004	−1.05 (−1.63, −0.47)	0.0004
Total NCV sum score	−0.32 (−0.43, −0.21)	<0.0001	−0.21 (−0.28, −0.14)	<0.0001	−0.33 (−0.44, −0.23)	<0.0001	−0.22 (−0.29, −0.15)	<0.0001
MNCV sum score	−0.38 (−0.50, −0.25)	<0.0001	−0.25 (−0.33, −0.16)	<0.0001	−0.40 (−0.52, −0.27)	<0.0001	−0.26 (−0.34, −0.18)	<0.0001
SNCV sum score	−0.26 (−0.38, −0.15)	<0.0001	−0.17 (−0.25, −0.09)	<0.0001	−0.27 (−0.39, −0.15)	<0.0001	−0.18 (−0.26, −0.09)	<0.0001

^a^Model 1: adjusted for sex and age at diagnosis

^b^Model 2: additionally adjusted for waist circumference, height, HbA_1c_, known diabetes duration, diabetes type, eGFR, total cholesterol, hypertension, CVD, lipid-lowering drugs and NSAIDs

**Table 4:** Associations of serum NFL with TDT

Outcome	Model 1^a^				Model 2^b^			
	Per 1-NPX		Per 1-SD		Per 1-NPX		Per 1-SD	
	β (95% CI)	*p* value	β (95% CI)	*p* value	β (95% CI)	*p* value	β (95% CI)	*p* value
Cold TDT (thenar eminence)	−0.01 (−0.14, 0.12)	0.883	−0.006 (−0.09, 0.08)	0.883	0.02 (−0.10, 0.14)	0.767	0.01 (−0.07, 0.09)	0.767
Cold TDT (dorsal foot)	−0.07 (−0.70, 0.54)	0.804	−0.05 (−0.46, 0.36)	0.804	−0.30 (−0.82, 0.21)	0.244	−0.20 (−0.55, 0.14)	0.244
Warm TDT (thenar eminence)	0.02 (−0.12, 0.16)	0.790	0.01 (−0.08, 0.10)	0.790	0.14 (0.02, 0.26)	0.023	0.09 (0.01, 0.17)	0.023
Warm TDT (dorsal foot)	0.69 (−0.22, 1.62)	0.139	0.46 (−0.15, 1.07)	0.139	1.22 (0.39, 2.04)	0.004	0.81 (0.26, 1.36)	0.004

^a^Model 1: adjusted for sex and age at diagnosis

^b^Model 2: additionally adjusted for waist circumference, height, HbA_1c_, known diabetes duration, diabetes type, eGFR, total cholesterol, hypertension, CVD, use of lipid-lowering drugs and NSAIDs

### Exploratory analyses

ESM Fig. 6 shows that correlations between biomarkers were almost all positive (*r* values ranged between 0.1 and 0.5) and ESM Fig. 7 shows that correlations between most biomarkers and age, BMI, waist circumference and total cholesterol were predominantly positive. In contrast, correlations between biomarkers and eGFR were mainly negative. Most biomarkers, except cadherin (CDH)-15, secreted frizzled-related protein 1 (SFRP1) and signal recognition particle 14 kDa protein (SRP14), did not show differences in their expression levels when comparing individuals with and without DSPN (*p*>0.05) (ESM Table 3).

Associations of biomarkers with prevalent DSPN are reported in ESM Table 4. Only serum SFRP1 was positively associated with prevalent DSPN in model 1 and model 2 but this association did not remain significant after adjustment for multiple testing. β estimates for the associations of biomarkers with MNCVs, SNCVs and NCV sum scores are shown in ESM Fig. 8 (β estimates by 1-NPX increase) and in ESM Fig. 9 (β estimates by 1-SD increase). Most associations were inverse. After full adjustment for covariates, eight biomarkers were associated with MNCV in at least one motor nerve and six biomarkers were associated with SNCV in at least one sensory nerve (*p*<0.05). In particular, CDH17 and disintegrin and metalloproteinase domain-containing protein 15 (ADAM15) showed inverse associations with the three sensory nerves investigated. Proline-rich Akt 1 substrate 1 (AKT1S1) was the only biomarker inversely associated with MNCV and SNCV. However, these associations were abolished when multiple testing was taken into account.

## Discussion

The results of this cross-sectional study in individuals recently diagnosed with type 1 and type 2 diabetes from the GDS baseline cohort demonstrated associations between higher serum NFL levels and prevalent DSPN. In addition, we showed the association of higher serum NFL levels with large myelinated fibre dysfunction, evident by slower MNCV and SNCV as well as a lower SNAP. Moreover, higher serum NFL levels were associated with elevated TDTs to warm rather than cold stimuli, indicating that small unmyelinated C-fibres, rather than thinly myelinated Aδ-fibres, contributed to this relationship. These associations were independent of age and other covariables, robust to sensitivity analyses, and aligned with our hypothesis that serum NFL is a promising biomarker to indicate early peripheral nerve dysfunction due to DSPN. However, this study did not reveal any associations between other neurological biomarkers and DSPN in exploratory analyses.

Our study is the first to show that high serum NFL levels are associated with an almost fourfold higher prevalence of DSPN. We measured serum NFL with a sensitive method and defined DSPN using the Toronto Consensus criteria. Additionally, we adjusted the associations for relevant confounders. When analysing NCS data separately, associations were more pronounced in the peroneal motor and sural sensory nerves than in the median and ulnar motor and sensory nerves. This pattern indicates a relatively higher degree of myelin damage and more intense axonal damage in lower limb nerves than in upper limb nerves. Such a finding is plausible for the following reasons: (1) the longer lower limb axons are more vulnerable to injury than upper limb axons [4]; (2) substantial evidence suggests that the earliest nerve damage occurs in the sural nerve early after diabetes diagnosis [42]; and (3) electrophysiological measures acquired in the sural sensory and peroneal motor nerves are considered the most sensitive NCS by which to detect early large fibre dysfunction in diabetes [43] and represent the first-line tests by the American Academy of Neurology [44].

Our findings considerably extend those of a previous epidemiological study reporting inverse correlations between serum NFL and NCV in the peroneal motor but not the sural sensory nerve in individuals with type 2 diabetes [29]. However, that study reported only Spearman’s correlations and did not estimate the associations between serum NFL and NCVs in multivariable models, possibly introducing bias due to confounding. In contrast, our study adjusted for multiple confounders. In addition, the previous study’s participants included only individuals with type 2 diabetes who were relatively older (age 35–85 years) and had a longer known diabetes duration (up to 3 years) compared with our study, which included participants with type 1 diabetes and type 2 diabetes, who were relatively younger (median age 47.7 years) and had a median known diabetes duration of 6 months. Therefore, our study provides the first comprehensive analysis of the associations between serum NFL and nerve function in individuals recently diagnosed with diabetes.

Notably, the observed associations between serum NFL and sural sensory nerve function are consistent with sural nerve biopsies indicating a positive correlation between higher serum NFL levels and axonal loss in older individuals with peripheral neuropathy of different aetiologies (6% diabetic) [45]. Thus, both electrophysiological and pathological findings reinforce the utility of serum NFL in quantifying axonal degeneration.

Though NFL is known as a biomarker of axonal loss, we detected more robust associations with NCV (typically regarded as an indicator of myelin damage) than with SNAP (typically regarded as an indicator of axonal loss). Evidence indicates that demyelination can exist in people with diabetes with and without symptomatic DSPN, but the axonal loss is linked to the appearance of symptoms [46]. Since only 14 out of 66 individuals with DSPN in our sample were symptomatic, this low prevalence of symptomatic DSPN might explain our study’s less pronounced associations between NFL and SNAP compared with the more pronounced associations with NCV. Nevertheless, the observed associations of NFL with both NCV and SNAP, though to a different extent, could suggest that NFL can be considered a biomarker of overall nerve injury (axonal loss and demyelination) rather than solely axonal loss. Others have shown increased NFL levels in individuals with polyneuropathies of different aetiologies irrespective of the associated pattern (demyelinating, axonal or both) [22], strengthening the suggestion that increased systemic NFL levels may be an indicator of either axonal damage, demyelination or both.

Associations between serum NFL and nerve conduction were more robust and consistent than associations between NFL and sensory nerve function, although large nerve fibres represent only a tiny fraction of nerve fibres. This difference is likely due to the abundant expression of NFL in large fibres where it is needed to increase the axonal diameter and speed up nerve conduction [10], unlike small sensory nerves with minimal nerve conduction that do not express high NFL levels. Another reason for this difference is that NCSs represent a more objective and robust instrument to estimate DSPN than QST [47]. Consequently, NCS-driven measures of nerve function in large nerves might be more relevant in this context than QST-driven measures in small nerves. In contrast to our study, which found associations of serum NFL with TDT to warm but not cold stimuli, a previous study measuring serum NFL with the Simoa Technology showed correlations with both warm and cold stimuli [29], although that study reported only non-adjusted correlations.

This present study demonstrated higher age-independent serum NFL levels in individuals with DSPN than in those without DSPN shortly after a diabetes diagnosis. A previous study in individuals with known diabetes duration of less than 3 years did not find a difference in serum NFL levels between individuals with and without DSPN [29]. However, the same study found a difference in serum NFL levels between individuals with DSPN and healthy control individuals. In another study, circulatory *NFL* mRNA was higher in individuals with DSPN than in those without DSPN [30]. It is worth noting that elevated serum NFL levels have been reported in peripheral neuropathies other than DSPN [13, 15, 17, 22, 25].

Substantial evidence associates higher serum NFL levels with various neurodegenerative diseases of the peripheral and central nervous systems [12]. Therefore, serum NFL is not a DSPN-specific biomarker and cannot be used as a biomarker for the diagnosis of DSPN. However, if the association between serum NFL and DSPN reported in our study is validated in other studies, future research should investigate the clinical utility of serum NFL as a biomarker for DSPN monitoring, given that a periodic serum NFL measurement is non-invasive and can be accessible in daily clinical care. Additionally, future studies might then investigate the potential of serum NFL as a candidate surrogate endpoint in phase II trials evaluating new therapies for DSPN, given that laboratory-measured biomarkers are objective, reproducible and not subject to inter-observer variability.

Exploratory analysis revealed inverse associations between some neurological biomarkers (e.g. ADAM15, involved in wound healing) and NCVs; these associations were abolished after adjustment for multiple testing, likely because Bonferroni correction is a conservative approach. It is also plausible that biomarkers reflecting nerve repair processes and axon development have a limited role in recent-onset diabetes. Thus, future studies, including participants with more prolonged diabetes, may reveal more biomarkers associated with nerve injury.

This study has several strengths. First, we included relatively young individuals with a median known diabetes duration of 6 months and good overall health status. This selection allowed analyses of associations without the confounding effect of late diabetes-related and ageing-related comorbidities. Second, neurophysiological testing targeted both large and small fibre functions. Third, our statistical analysis was comprehensively adjusted for covariates and associations were assessed for different outcomes modelled as a binary variable, continuous nerve function in single nerves and sum scores. Our study includes the following limitations: (1) the cross-sectional design precludes us from knowing the predictive value of serum NFL; (2) the inclusion of individuals with well-controlled diabetes for whom the extent of nerve damage is lower than individuals with less-controlled diabetes; (3) the predominance of subclinical DSPN prevents us from assessing the associations between serum NFL and DSPN stages; and (4) the lack of statistical power to stratify the analyses by diabetes type. However, as the interaction between serum NFL and diabetes type was not significant, there was no evidence for a difference in the observed associations between type 1 and type 2 diabetes. In addition, biomarkers were measured with the highly sensitive proximity extension assay technology. Though protein levels were expressed as NPX values rather than absolute concentrations, this aspect did not impact our findings. Finally, our study population consists of mainly German individuals with short known diabetes duration. Thus, our findings cannot be generalisable to other ethnicities and individuals with longer diabetes duration.

### Conclusion

Our study indicates that higher serum NFL levels are associated with prevalent DSPN and nerve dysfunction in recently diagnosed diabetes. We advocate serum NFL as a novel blood-based biomarker of nerve injury in DSPN. However, the potential clinical utility of serum NFL as a monitoring biomarker for DSPN in routine clinical practice and as a biomarker in clinical trials needs to be investigated in future studies.

## Supplementary information

ESM (PDF 1818 kb)

## Data Availability

The data are subject to national data protection laws. Therefore, data cannot be made freely available in a public repository. However, data can be requested through an individual project agreement with the Steering Committee of the GDS (speaker: M. Roden, michael.roden@ddz.de).

## Abbreviations

ADAM15: Disintegrin and metalloproteinase domain-containing protein 15
CDH: Cadherin
DSPN: Diabetic sensorimotor polyneuropathy
GDS: German Diabetes Study
MNCV: Motor nerve conduction velocity
NCS: Nerve conduction study
NCV: Nerve conduction velocity
NFL: Neurofilament light chain
NPX: Normalised protein expression
NSAID: Non-steroidal anti-inflammatory drug
QST: Quantitative sensory testing
SFRP1: Secreted frizzled-related protein 1
SNAP: Sensory nerve action potential
SNCV: Sensory nerve conduction velocity
TDT: Thermal detection threshold
