# Supplementary material for: Serum neurofilament light chain: a novel biomarker for early diabetic sensorimotor polyneuropathy
Source: Diabetologia. 2022 Dec 6;66(3):579–89. doi: 10.1007/s00125-022-05846-8 (PMC9892145; doi:10.1007/s00125-022-05846-8)
Supplement: Supplementary file 1 — (PDF 1818 kb) [file 125_2022_5846_MOESM1_ESM.pdf]

Supplementary Material

Serum neurofilament light chain: A novel biomarker for early diabetic sensorimotor polyneuropathy

Haifa Maalmi[1,2], Alexander Strom[1,2], Agnese Petrera[2,3], Stefanie M. Hauck[2,3], Klaus Strassburger[2,4], Oliver Kuss[2,4,5], Oana-Patricia Zaharia[1,2,6], Gidon J. Bönhof[1,2,6], Wolfgang Rathmann[2,4], Sandra Trenkamp[1,2], Volker Burkart[1,2], Julia Szendroedi[1,2,7,8], Dan Ziegler[1,2,6], Michael Roden[1,2,6], Christian Herder[1,2,6] and the GDS Group

1. Institute for Clinical Diabetology, German Diabetes Center (Deutsches Diabetes-Zentrum/DDZ), Leibniz Center for Diabetes Research at Heinrich Heine University Düsseldorf, Düsseldorf, Germany

2. German Center for Diabetes Research (DZD), München-Neuherberg, Germany

3. Metabolomics and Proteomics Core, Helmholtz Zentrum München, German Research Center for Environmental Health (GmbH), Neuherberg, Germany

4. Institute for Biometrics and Epidemiology, German Diabetes Center (Deutsches Diabetes-Zentrum/DDZ), Düsseldorf, Germany

5. Centre for Health and Society, Medical Faculty, Heinrich Heine University, Düsseldorf, Germany

6. Department of Endocrinology and Diabetology, Medical Faculty and University Hospital Düsseldorf, Heinrich Heine University Düsseldorf, Düsseldorf, Germany

7. Department of Endocrinology, Diabetology, Metabolism and Clinical Chemistry, Heidelberg University Hospital, Heidelberg, Germany

8. Institute for Diabetes and Cancer (IDC) & Joint Heidelberg–IDC Translational Diabetes Program, Helmholtz Center Munich, München-Neuherberg, Germany

Dan Ziegler, Michael Roden and Christian Herder contributed equally to this study.

The GDS Group consists of H. Al-Hasani, V. Burkart, A. E. Buyken, G. Geerling, C. Herder, A. Icks, K. Jandeleit-Dahm, J. Kotzka, O. Kuss, E. Lammert, W. Rathmann, V. Schrauwen-Hinderling, J. Szendroedi, S. Trenkamp, D. Ziegler and M. Roden (speaker).

ESM Table 1. List of the 92 biomarkers in the OLINK NEURO EXPLORATORY panel and assay characteristics

Assay	Full name	Uniprot ID	Olink ID	LOD (NPX)	Missing Data freq (% of samples < LOD)	intra-assay (%)	inter-assay (%)	Exclusion
**AARSD1**	Alanyl-tRNA editing protein Aarsd1	Q9BTE6	OID05202	1.82569	11%	6.05	16.16	
**ABHD14B**	Protein ABHD14B	Q96IU4	OID05201	0.81444	5%	9.90	33.62	§§
**ADAM15**	Disintegrin and metalloproteinase domain-containing protein 15	Q13444	OID05162	2.12965	0%	1.15	2.33	
**ADGRB3**	Adhesion G protein-coupled receptor B3	O60242	OID05521	1.36213	0%	2.25	4.39	
**AKT1S1**	Proline-rich AKT1 substrate 1	Q96B36	OID05192	0.81980	0%	3.57	8.47	
**ANXA10**	Annexin A10	Q9UJ72	OID05147	1.42621	0%	2.53	6.97	
**AOC1**	Amiloride-sensitive amine oxidase (copper-containing)	P19801	OID05212	1.45281	43%	10.63	25.26	§
**ASGR1**	Asialoglycoprotein receptor 1	P07306	OID05183	0.56951	0%	2.61	5.60	
**ATP6V1F**	V-type proton ATPase subunit F	Q16864	OID05143	0.77517	57%	15.37	26.25	§
**BST2**	Bone marrow stromal antigen 2	Q10589	OID05140	1.64665	0%	2.03	3.75	
**CARHSP1**	Calcium-regulated heat-stable protein 1	Q9Y2V2	OID05198	2.48383	79%	10.04	13.54	§
**CCL27**	C-C motif chemokine 27	Q9Y4X3	OID05190	0.84066	0%	2.25	3.65	
**CD302**	CD302 antigen	Q8IX05	OID05152	0.96903	0%	1.36	2.38	
**CD33**	Myeloid cell surface antigen CD33	P20138	OID05161	0.50305	0%	1.50	6.09	
**CD63**	CD63 antigen	P08962	OID05222	1.34742	0%	0.82	3.85	
**CDH15**	Cadherin-15	P55291	OID05134	1.92944	0%	2.48	3.93	
**CDH17**	Cadherin-17	Q12864	OID05138	0.75868	0%	2.21	4.21	
**CEACAM3**	Carcinoembryonic antigen-related cell adhesion molecule 3	P40198	OID05164	1.52377	97%	46.05	59.82	§
**CETN2**	Centrin-2	P41208	OID05133	0.99720	39%	13.06	28.92	§
**CLSTN1**	Calsyntenin-1	O94985	OID05158	0.56127	9%	8.27	23.99	
**COL4A3BP**	Collagen type IV alpha-3-binding protein	Q9Y5P4	OID05185	2.00325	13%	5.16	9.92	
**CRADD**	Death domain-containing protein	P78560	OID05129	1.97500	0%	2.38	5.61	
**CRIP2**	Cysteine-rich protein 2	P52943	OID05154	3.24357	0%	2.78	5.72	
**CTF1**	Cardiotrophin-1	Q16619	OID05156	0.87927	95%	NA	NA	§

**DEFB4A**	Beta-defensin 4A	O15263	OID05200	-1.83046	0%	3.31	5.65	
**DPEP1**	Dipeptidase 1	P16444	OID05209	2.08402	0%	2.31	5.47	
**DPEP2**	Dipeptidase 2	Q9H4A9	OID05180	3.02144	1%	4.20	7.98	
**DSG3**	Desmoglein-3	P32926	OID05170	1.67650	0%	1.63	2.62	
**DUSP3**	Dual specificity protein phosphatase 3	P51452	OID05204	0.48394	66%	13.76	196.90	§
**ECE1**	Endothelin-converting enzyme 1	P42892	OID05132	2.27674	90%	10.25	15.97	§
**EIF4B**	Eukaryotic translation initiation factor 4B	P23588	OID05128	0.86764	0%	3.08	7.30	
**EPHA10**	Ephrin type-A receptor 10	Q5JZY3	OID05184	1.82915	90%	22.02	21.41	§
**EREG**	Proepiregulin	O14944	OID05151	1.03308	1%	3.30	6.15	
**FCAR**	Immunoglobulin alpha Fc receptor	P24071	OID05175	1.09115	0%	3.83	9.57	
**FGFR2**	Fibroblast growth factor receptor 2	P21802	OID05166	0.43136	0%	2.13	3.69	
**FHIT**	Bis(5'-adenosyl)-triphosphatase	P49789	OID05157	1.10833	47%	17.90	29.95	§
**FKBP5**	Peptidyl-prolyl cis-trans isomerase FKBP5	Q13451	OID05220	0.86250	0%	2.42	5.69	
**FKBP7**	Peptidyl-prolyl cis-trans isomerase FKBP7	Q9Y680	OID05146	1.33611	79%	18.38	31.84	§
**FUT8**	Alpha-(1.6)-fucosyltransferase	Q9BYC5	OID05195	1.15604	0%	2.38	4.64	
**GBP2**	Guanylate-binding protein 2	P32456	OID05165	1.96856	98%	18.83	34.64	§
**GGT5**	Glutathione hydrolase 5 proenzyme	P36269	OID05221	1.68726	0%	1.91	3.64	
**GPNMB**	Transmembrane glycoprotein NMB	Q14956	OID05139	1.49694	0%	0.94	2.34	
**GSTP1**	Glutathione S-transferase P	P09211	OID05148	1.05669	94%	33.99	78.44	§
**HMOX2**	Heme oxygenase 2	P30519	OID05207	0.82644	0%	3.54	6.51	
**HSP90B1**	Endoplasmin	P14625	OID05159	1.41648	90%	24.40	33.22	§
**IFI30**	Gamma-interferon-inducible lysosomal thiol reductase	P13284	OID05174	-0.27131	0%	2.55	6.03	
**IFNL1**	Interferon lambda-1	Q8IU54	OID05127	1.92651	89%	18.75	25.45	§
**IKZF2**	Zinc finger protein Helios	Q9UKS7	OID05178	1.70866	91%	83.40	27.63	§
**IL15**	Interleukin-15	P40933	OID05169	0.48829	0%	3.64	5.03	
**IL32**	Interleukin-32	P24001	OID05194	1.43373	0%	2.59	6.03	
**IL3RA**	Interleukin-3 receptor subunit alpha	P26951	OID05211	0.87502	0%	8.05	16.82	
**ILKAP**	Integrin-linked kinase-associated serine/threonine phosphatase 2C	Q9H0C8	OID05215	2.35066	47%	6.58	7.39	§
**IMPA1**	Inositol monophosphatase 1	P29218	OID05153	1.47934	85%	14.06	25.84	§

**ING1**	Inhibitor of growth protein 1	Q9UK53	OID05217	1.97478	99%	11.42	24.50	§
**ISLR2**	mmunoglobulin superfamily containing leucine-rich repeat protein 2	Q6UXK2	OID05216	1.81788	6%	6.43	12.72	
**KIF1BP**	KIF1-binding protein	Q96EK5	OID05213	1.79928	11%	4.02	9.16	
**KIR2DL3**	Killer cell immunoglobulin-like receptor 2DL3	P43628	OID05149	1.14927	17%	5.70	13.48	
**KIRREL2**	Kin of IRRE-like protein 2	Q6UWL6	OID05188	1.86316	0%	4.45	9.94	
**KLB**	Beta-klotho	Q86Z14	OID05137	0.79081	0%	2.58	6.03	
**LEPR**	Leptin receptor	P48357	OID05160	2.01439	0%	1.60	2.96	
**LTBP3**	Latent-transforming growth factor beta-binding protein 3	Q9NS15	OID05522	1.98197	0%	4.96	8.06	
**MAD1L1**	Mitotic spindle assembly checkpoint protein MAD1	Q9Y6D9	OID05191	0.52484	0%	3.25	7.62	
**NAA10**	N-alpha-acetyltransferase 10	P41227	OID05168	2.41388	17%	5.50	9.02	
**NDRG1**	Protein NDRG1	Q92597	OID05163	1.36602	83%	11.13	24.09	§
**NEFL**	Neurofilament light polypeptide	P07196	OID05206	1.52833	0%	4.94	6.49	
**NPM1**	Nucleophosmin	P06748	OID05182	0.87659	0%	1.44	3.36	
**NXPH1**	Neurexophilin-1	P58417	OID05167	0.88243	86%	21.16	41.82	§
**PAEP**	Glycodelin	P09466	OID05189	1.21967	47%	20.88	32.98	§
**PFDN2**	Prefoldin subunit 2	Q9UHV9	OID05197	0.65553	17%	16.21	36.46	§§
**PHOSPHO1**	Phosphoethanolamine/phosphocholine phosphatase	Q8TCT1	OID05203	1.93143	0%	3.23	8.67	
**PLA2G10**	Group 10 secretory phospholipase A2	O15496	OID05177	1.71916	0%	2.24	3.77	
**PMVK**	Phosphomevalonate kinase	Q15126	OID05218	1.35322	0%	3.51	8.25	
**PPP3R1**	Calcineurin subunit B type 1	P63098	OID05214	0.56858	0%	1.85	3.01	
**PRTFDC1**	Phosphoribosyltransferase domain-containing protein 1	Q9NRG1	OID05176	2.57623	81%	14.58	18.71	§
**PSG1**	Pregnancy-specific beta-1-glycoprotein 1	P11464	OID05186	1.08791	3%	3.39	10.23	
**PSME1**	Proteasome activator complex subunit 1	Q06323	OID05187	0.97657	0%	1.42	4.05	
**PTPN1**	Tyrosine-protein phosphatase non-receptor type 1	P18031	OID05141	1.53237	0%	2.84	6.24	
**PTS**	6-pyruvoyl tetrahydrobiopterin synthase	Q03393	OID05193	1.36628	0%	3.31	5.10	
**RBKS**	Ribokinase	Q9H477	OID05144	0.68171	0%	1.84	4.16	
**RNF31**	E3 ubiquitin-protein ligase RNF31	Q96EP0	OID05208	1.09796	91%	55.06	48.62	§
**RPS6KB1**	Ribosomal protein S6 kinase beta-1	P23443	OID05150	1.03833	33%	11.50	21.79	§
**SFRP1**	Secreted frizzled-related protein 1	Q8N474	OID05171	2.19457	0%	2.89	3.86	

**SMOC1**	SPARC-related modular calcium-binding protein 1	Q9H4F8	OID05135	0.23440	0%	2.17	4.80	
**SNCG**	Gamma-synuclein	O76070	OID05210	1.62108	5%	6.50	10.58	
**SRP14**	Signal recognition particle 14 kDa protein	P37108	OID05142	0.76122	0%	1.60	2.22	
**TBCB**	Tubulin-folding cofactor B	Q99426	OID05181	1.08991	0%	3.72	6.13	
**TDGF1**	Teratocarcinoma-derived growth factor 1	P13385	OID05131	1.71268	46%	5.99	9.14	§
**TNFRSF13C**	Tumor necrosis factor receptor superfamily member 13C	Q96RJ3	OID05205	1.49207	73%	17.37	27.43	§
**TPPP3**	Tubulin polymerization-promoting protein family member 3	Q9BW30	OID05196	1.34300	40%	12.85	18.90	§
**UBE2F**	NEDD8-conjugating enzyme UBE2F	Q969M7	OID05179	0.06455	84%	NA	NA	§
**VSTM1**	V-set and transmembrane domain-containing protein 1	Q6UX27	OID05199	1.12514	6%	6.46	11.21	
**WWP2**	NEDD4-like E3 ubiquitin-protein ligase	O00308	OID05219	1.83647	0%	2.01	3.74	

Abbreviations: LOD, limit of detection; NA, not applicable (NPX of control measurements below LOD); NPX, normalised protein expression values. NEFL refers to neurofilament light polypeptide or neurofilament light chain or NFL.

The calculation of inter-and intra-assay CVs and the percentage of samples below the LOD was based on measurements of 503 participants recruited consecutively from 2005 to 2011.

§ Excluded from analysis because of missing data for ≥25%.

§§ Excluded from analysis because inter-assay CV >25%

ESM Table 2. Correlations of serum NFL with demographic and metabolic variables

	Non-adjusted correlation		Age-adjusted correlation	
**Variable**	**r**	***p***	**r**	***p***
Age at diagnosis	0.61	<0.0001	-	-
BMI	0.13	0.007	-0.14	0.005
Waist circumference	0.13	0.006	-0.13	0.008
Height	-0.15	0.002	-0.03	0.546
HbA1c	0.05	0.303	0.09	0.049
Total cholesterol	0.13	0.008	-0.05	0.299
eGFR	-0.36	<0.0001	-0.004	0.928

BMI, body mass index; eGFR, estimated glomerular filtration rate

ESM Table 3. Expression levels of 60 neurological biomarkers according to DSPN status

Biomarker	DSPN (n=66)	No DSPN (n=357)
AARSD1	2.3 (2.0, 2.7)	2.3 (2.0, 2.7)
ADAM15	8.8 (8.6, 9.1)	8.8 (8.6, 9.1)
ADGRB3	5.9 (5.6, 6.1)	5.8 (5.6, 6.1)
AKT1S1	2.6 (2.2, 3.1)	2.7 (2.4, 3.0)
ANXA10	2.9 (2.4, 3.3)	2.8 (2.4, 3.6)
ASGR1	4.3 (4.1, 4.7)	4.4 (4.1, 4.6)
BST2	5.8 (5.5, 6.1)	5.8 (5.5, 6.1)
CCL27	5.0 (4.8, 5.3)	5.1 (4.8, 5.3)
CD302	7.2 (6.9, 7.4)	7.1 (6.8, 7.3)
CD33	5.1 (4.2, 5.4)	5.2 (4.3, 5.5)
CD63	8.3 (8.1, 8.6)	8.4 (8.1, 8.6)
CDH15	8.0 (7.2, 8.8)	7.6 (7.0, 8.3)
CDH17	5.9 (5.7, 6.3)	6.0 (5.5, 6.3)
CLSTN1	0.8 (0.6, 1.0)	0.8 (0.7, 1.0)
COL4A3BP	2.3 (2.1, 2.6)	2.4 (2.2, 2.6)
CRADD	4.6 (4.3, 5.0)	4.6 (4.4, 5.2)
CRIP2	5.4 (5.1, 5.6)	5.4 (5.1, 5.6)
DEFB4A	4.9 (3.9, 5.7)	4.6 (3.5, 5.5)
DPEP1	4.0 (3.7, 4.3)	4.0 (3.7, 4.3)
DPEP2	3.8 (3.6, 4.0)	3.8 (3.7, 4.0)
DSG3	6.7 (6.4, 7.0)	6.7 (6.4, 7.0)
EIF4B	4.1 (3.5, 4.7)	4.3 (3.7, 4.8)
EREG	4.5 (4.0, 5.3)	4.6 (4.1, 5.2)
FCAR	2.5 (2.3, 2.8)	2.6 (2.2, 2.9)
FGFR2	5.1 (5.0, 5.2)	5.1 (4.9, 5.3)
FKBP5	4.0 (3.4, 4.5)	4.0 (3.6, 4.6)
FUT8	4.6 (4.3, 5.0)	4.7 (4.4, 5.1)
GGT5	5.7 (5.6, 5.9)	5.7 (5.6, 5.9)
GPNMB	7.9 (7.8, 8.0)	7.9 (7.8, 8.0)
HMOX2	2.9 (2.6, 3.2)	2.9 (2.7, 3.2)
IFI30	3.4 (3.1, 3.8)	3.6 (3.2, 4.0)
IL15	3.4 (3.2, 3.6)	3.3 (3.2, 3.5)
IL32	5.3 (5.2, 5.7)	5.5 (5.2, 5.8)
IL3RA	1.9 (1.7, 2.2)	1.9 (1.6, 2.2)
ISLR2	2.4 (2.0, 2.6)	2.4 (2.2, 2.7)
KIF1BP	2.2 (1.9, 2.6)	2.3 (2.0, 2.6)
KIR2DL3	1.7 (1.4, 2.1)	1.7 (1.3, 2.2)
KIRREL2	2.9 (2.6, 3.0)	2.8 (2.6, 3.0)
KLB	3.6 (3.2, 4.0)	3.6 (3.1, 3.9)
LEPR	5.6 (5.4, 5.8)	5.6 (5.4, 5.8)
LTBP3	3.1 (3.0, 3.4)	3.1 (2.8, 3.4)

Biomarker	DSPN (n=66)	No DSPN (n=357)
MAD1L1	3.1 (2.9, 3.5)	3.2 (2.9, 3.5)
NAA10	2.8 (2.5, 3.3)	2.9 (2.5, 3.3)
NPM1	5.2 (4.7, 5.7)	5.3 (5.0, 5.9)
PHOSPHO1	3.5 (3.3, 3.8)	3.4 (3.2, 3.7)
PLA2G10	5.5 (5.0, 5.9)	5.6 (5.1, 5.9)
PMVK	3.4 (2.9, 3.9)	3.4 (3.0, 4.0)
PPP3R1	3.8 (3.6, 4.1)	3.8 (3.6, 4.0)
PSG1	4.0 (3.4, 4.6)	3.7 (3.1, 4.4)
PSME1	4.2 (4.0, 4.4)	4.2 (4.0, 4.4)
PTPN1	4.8 (4.4, 5.2)	4.8 (4.5, 5.3)
PTS	4.4 (4.0, 4.9)	4.3 (3.9, 5.0)
RBKS	5.9 (5.5, 6.3)	5.8 (5.5, 6.2)
SFRP1	5.7 (5.3, 6.1)	5.5 (5.1, 5.9)
SMOC1	5.1 (4.8, 5.4)	5.0 (4.7, 5.3)
SNCG	2.8 (2.4, 3.2)	2.7 (2.3, 3.2)
SRP14	4.7 (4.1, 5.5)	5.1 (4.5, 5.8)
TBCB	4.0 (3.3, 4.7)	4.2 (3.6, 4.7)
VSTM1	2.0 (1.6, 2.4)	2.0 (1.5, 2.3)
WWP2	4.7 (4.6, 5.2)	4.9 (4.6, 5.3)

Data are expressed as median (25th, 75th percentiles) of NPX values

Biomarkers abbreviations are specified in ESM Table1.

ESM Table 4. Risk ratios (RR) and 95% confidence intervals (CIs) for the associations between neurological biomarkers and prevalent DSPN

	**Model 1**						**Model 2**					
	Per 1-NPX			***Per 1-SD***			***Per 1-NPX***			***Per 1-SD***		
**Biomarker**	**RR**	**95% CI**	***p***	**RR**	**95% CI**	***p***	**RR**	**95% CI**	***p***	**RR**	**95% CI**	***p***
AARSD1	1.13	(0.82, 1.57)	0.462	1.08	(0.88, 1.34)	0.462	1.10	(0.80, 1.53)	0.554	1.07	(0.86, 1.32)	0.554
ADAM15	1.37	(0.79, 2.36)	0.261	1.13	(0.91, 1.41)	0.261	1.47	(0.86, 2.50)	0.159	1.17	(0.94, 1.44)	0.159
ADGRB3	1.01	(0.59, 1.71)	0.976	1.00	(0.82, 1.23)	0.976	1.15	(0.69, 1.92)	0.596	1.06	(0.87, 1.29)	0.596
AKT1S1	1.04	(0.72, 1.50)	0.850	1.03	(0.79, 1.32)	0.85	1.02	(0.71, 1.48)	0.907	1.02	(0.78, 1.31)	0.907
ANXA10	1.04	(0.82, 1.31)	0.754	1.04	(0.83, 1.28)	0.754	1.06	(0.83, 1.35)	0.667	1.05	(0.84, 1.32)	0.667
ASGR1	1.07	(0.53, 2.13)	0.856	1.02	(0.79, 1.33)	0.856	0.82	(0.41, 1.63)	0.573	0.93	(0.72, 1.2)	0.573
BST2	0.98	(0.61, 1.57)	0.922	0.99	(0.8, 1.23)	0.922	0.91	(0.58, 1.45)	0.702	0.96	(0.78, 1.18)	0.702
CCL27	0.94	(0.48, 1.87)	0.870	0.98	(0.77, 1.24)	0.87	1.07	(0.55, 2.10)	0.837	1.02	(0.81, 1.29)	0.837
CD302	1.54	(0.93, 2.55)	0.096	1.19	(0.97, 1.45)	0.096	1.53	(0.93, 2.53)	0.094	1.19	(0.97, 1.45)	0.094
CD33	0.90	(0.73, 1.10)	0.303	0.90	(0.73, 1.1)	0.303	0.95	(0.77, 1.18)	0.658	0.95	(0.77, 1.18)	0.658
CD63	0.84	(0.46, 1.51)	0.556	0.94	(0.77, 1.15)	0.556	0.87	(0.49, 1.55)	0.638	0.95	(0.78, 1.16)	0.638
CDH15	0.96	(0.76, 1.21)	0.709	0.95	(0.75, 1.22)	0.709	0.91	(0.73, 1.14)	0.419	0.91	(0.71, 1.15)	0.419
CDH17	1.24	(0.84, 1.82)	0.284	1.13	(0.9, 1.41)	0.284	1.30	(0.89, 1.91)	0.176	1.16	(0.93, 1.45)	0.176
CLSTN1	0.68	(0.34, 1.38)	0.290	0.83	(0.6, 1.17)	0.29	0.64	(0.31, 1.31)	0.222	0.81	(0.58, 1.14)	0.222
COL4A3BP	0.89	(0.49, 1.63)	0.709	0.96	(0.77, 1.19)	0.709	0.94	(0.52, 1.69)	0.834	0.98	(0.79, 1.21)	0.834
CRADD	1.02	(0.72, 1.44)	0.903	1.01	(0.81, 1.28)	0.903	1.05	(0.75, 1.47)	0.787	1.03	(0.82, 1.29)	0.787
CRIP2	0.80	(0.43, 1.49)	0.489	0.91	(0.7, 1.18)	0.489	0.82	(0.42, 1.62)	0.570	0.92	(0.69, 1.23)	0.570
DEFB4A	1.09	(0.93, 1.27)	0.300	1.12	(0.9, 1.38)	0.3	1.06	(0.91, 1.24)	0.437	1.09	(0.88, 1.33)	0.437
DPEP1	0.95	(0.62, 1.44)	0.804	0.97	(0.79, 1.2)	0.804	1.10	(0.72, 1.69)	0.664	1.05	(0.85, 1.3)	0.664
DPEP2	0.64	(0.30, 1.37)	0.253	0.89	(0.72, 1.09)	0.253	0.72	(0.32, 1.64)	0.435	0.92	(0.73, 1.14)	0.435

	Model 1						Model 2					
	Per 1-NPX			***Per 1-SD***			***Per 1-NPX***			***Per 1-SD***		
**Biomarker**	**RR**	**95% CI**	***p***	**RR**	**95% CI**	***p***	**RR**	**95% CI**	***p***	**RR**	**95% CI**	***p***
DSG3	0.79	(0.45, 1.37)	0.398	0.91	(0.72, 1.14)	0.398	0.84	(0.50, 1.42)	0.518	0.93	(0.75, 1.16)	0.518
EIF4B	0.88	(0.68, 1.15)	0.360	0.88	(0.67, 1.15)	0.36	0.93	(0.72, 1.22)	0.613	0.93	(0.71, 1.22)	0.613
EREG	0.98	(0.79, 1.22)	0.855	0.98	(0.79, 1.22)	0.855	0.99	(0.81, 1.22)	0.933	0.99	(0.81, 1.22)	0.933
FCAR	0.94	(0.61, 1.45)	0.779	0.97	(0.80, 1.18)	0.779	0.96	(0.63, 1.47)	0.867	0.98	(0.81, 1.19)	0.867
FGFR2	1.04	(0.49, 2.22)	0.923	1.01	(0.81, 1.25)	0.923	0.85	(0.40, 1.80)	0.670	0.95	(0.77, 1.18)	0.670
FKBP5	0.90	(0.68, 1.19)	0.476	0.92	(0.74, 1.15)	0.476	0.96	(0.72, 1.26)	0.753	0.97	(0.78, 1.20)	0.753
FUT8	0.85	(0.56, 1.30)	0.456	0.92	(0.73, 1.15)	0.456	0.87	(0.57, 1.33)	0.522	0.93	(0.74, 1.17)	0.522
GGT5	1.45	(0.70, 2.98)	0.317	1.11	(0.90, 1.36)	0.317	1.43	(0.73, 2.81)	0.300	1.11	(0.91, 1.34)	0.300
GPNMB	1.24	(0.32, 4.72)	0.755	1.04	(0.83, 1.30)	0.755	1.44	(0.40, 5.21)	0.581	1.06	(0.85, 1.32)	0.581
HMOX2	0.87	(0.55, 1.37)	0.543	0.93	(0.74, 1.18)	0.543	0.89	(0.57, 1.41)	0.621	0.94	(0.74, 1.19)	0.621
IFI30	0.69	(0.44, 1.07)	0.095	0.83	(0.66, 1.03)	0.095	0.65	(0.41, 1.02)	0.059	0.8	(0.64, 1.01)	0.059
IL15	1.27	(0.51, 3.15)	0.608	1.07	(0.83, 1.38)	0.608	1.36	(0.56, 3.30)	0.502	1.09	(0.85, 1.40)	0.502
IL32	1.01	(0.64, 1.59)	0.961	1.01	(0.79, 1.27)	0.961	1.12	(0.73, 1.70)	0.605	1.06	(0.85, 1.32)	0.605
IL3RA	0.84	(0.42, 1.65)	0.609	0.94	(0.74, 1.19)	0.609	0.99	(0.50, 1.97)	0.977	1.00	(0.78, 1.27)	0.977
ISLR2	0.74	(0.42, 1.30)	0.295	0.88	(0.70, 1.12)	0.295	0.79	(0.46, 1.37)	0.406	0.91	(0.72, 1.14)	0.406
KIF1BP	0.99	(0.61, 1.62)	0.983	1.00	(0.79, 1.26)	0.983	1.06	(0.65, 1.74)	0.809	1.03	(0.81, 1.30)	0.809
KIR2DL3	1.01	(0.74, 1.37)	0.970	1.00	(0.81, 1.24)	0.97	1.00	(0.75, 1.35)	0.975	1.00	(0.82, 1.23)	0.975
KIRREL2	1.05	(0.55, 1.99)	0.880	1.02	(0.83, 1.25)	0.88	1.17	(0.62, 2.22)	0.619	1.05	(0.86, 1.29)	0.619
KLB	1.01	(0.71, 1.44)	0.959	1.01	(0.81, 1.25)	0.959	0.99	(0.71, 1.37)	0.946	0.99	(0.81, 1.21)	0.946
LEPR	1.18	(0.53, 2.61)	0.688	1.05	(0.81, 1.37)	0.688	1.82	(0.77, 4.26)	0.171	1.21	(0.92, 1.60)	0.171
LTBP3	1.16	(0.80, 1.68)	0.424	1.08	(0.90, 1.28)	0.424	1.15	(0.76, 1.74)	0.523	1.07	(0.87, 1.30)	0.523
MAD1L1	0.82	(0.49, 1.38)	0.456	0.92	(0.73, 1.15)	0.456	0.88	(0.52, 1.47)	0.618	0.94	(0.75, 1.18)	0.618
NAA10	1.00	(0.69, 1.47)	0.980	1.00	(0.81, 1.24)	0.98	1.08	(0.74, 1.56)	0.690	1.04	(0.85, 1.29)	0.69
NPM1	0.78	(0.55, 1.09)	0.147	0.85	(0.68, 1.06)	0.147	0.83	(0.60, 1.16)	0.273	0.89	(0.71, 1.10)	0.273

	Model 1						Model 2					
	Per 1-NPX			***Per 1-SD***			***Per 1-NPX***			***Per 1-SD***		
**Biomarker**	**RR**	**95% CI**	***p***	**RR**	**95% CI**	***p***	**RR**	**95% CI**	***p***	**RR**	**95% CI**	***p***
PHOSPHO1	1.49	(0.69, 3.21)	0.307	1.14	(0.88, 1.48)	0.307	1.54	(0.74, 3.21)	0.244	1.16	(0.90, 1.48)	0.244
PLA2G10	0.95	(0.69, 1.30)	0.739	0.96	(0.78, 1.20)	0.739	0.97	(0.66, 1.43)	0.880	0.98	(0.75, 1.28)	0.880
PMVK	0.92	(0.70, 1.22)	0.576	0.94	(0.75, 1.17)	0.576	0.96	(0.74, 1.25)	0.769	0.97	(0.78, 1.20)	0.769
PPP3R1	1.12	(0.48, 2.60)	0.797	1.03	(0.80, 1.33)	0.797	1.05	(0.50, 2.21)	0.894	1.02	(0.81, 1.27)	0.894
PSG1	1.17	(0.92, 1.49)	0.193	1.20	(0.91, 1.57)	0.193	1.20	(0.97, 1.50)	0.095	1.23	(0.96, 1.58)	0.095
PSME1	1.08	(0.64, 1.84)	0.764	1.04	(0.83, 1.30)	0.764	1.05	(0.60, 1.83)	0.872	1.02	(0.80, 1.30)	0.872
PTPN1	0.95	(0.69, 1.31)	0.755	0.97	(0.78, 1.20)	0.755	0.98	(0.72, 1.33)	0.891	0.99	(0.80, 1.21)	0.891
PTS	1.10	(0.83, 1.46)	0.522	1.08	(0.85, 1.37)	0.522	0.96	(0.70, 1.32)	0.823	0.97	(0.75, 1.26)	0.823
RBKS	1.03	(0.71, 1.50)	0.880	1.02	(0.80, 1.30)	0.88	0.92	(0.61, 1.40)	0.707	0.95	(0.73, 1.24)	0.707
SFRP1	1.53	(1.07, 2.20)	0.021	1.30	(1.04, 1.63)	0.021	1.50	(1.01, 2.23)	0.045	1.28	(1.01, 1.64)	0.045
SMOC1	1.40	(0.85, 2.32)	0.189	1.18	(0.92, 1.50)	0.189	1.34	(0.81, 2.24)	0.257	1.15	(0.90, 1.48)	0.257
SNCG	1.19	(0.82, 1.72)	0.372	1.13	(0.86, 1.48)	0.372	1.08	(0.72, 1.62)	0.723	1.05	(0.79, 1.42)	0.723
SRP14	0.81	(0.63, 1.03)	0.087	0.82	(0.65, 1.03)	0.087	0.84	(0.67, 1.06)	0.148	0.85	(0.68, 1.06)	0.148
TBCB	0.96	(0.72, 1.28)	0.782	0.97	(0.75, 1.24)	0.782	0.97	(0.77, 1.21)	0.755	0.97	(0.80, 1.17)	0.755
VSTM1	1.29	(0.90, 1.84)	0.160	1.16	(0.94, 1.42)	0.16	1.28	(0.89, 1.84)	0.185	1.15	(0.94, 1.42)	0.185
WWP2	0.84	(0.51, 1.37)	0.476	0.92	(0.72, 1.17)	0.476	0.91	(0.58, 1.42)	0.678	0.95	(0.77, 1.19)	0.678

Biomarkers abbreviations are specified in ESM Table 1; CI, confidence interval; DSPN, diabetic sensorimotor polyneuropathy; NPX, Normalized Protein eXpression; RR, risk ratio; SD, standard deviation.

Model 1: adjusted for age and sex.

Model 2: adjusted for model 1+ waist circumference, height, HbA1C, known diabetes duration, diabetes type, eGFR, total cholesterol, hypertension, CVD, use of lipid-lowering drugs and NSAIDs.

Bonferroni-corrected $p<0.0008$ indicates significant associations.

ESM Fig.1. Flowchart of the study population

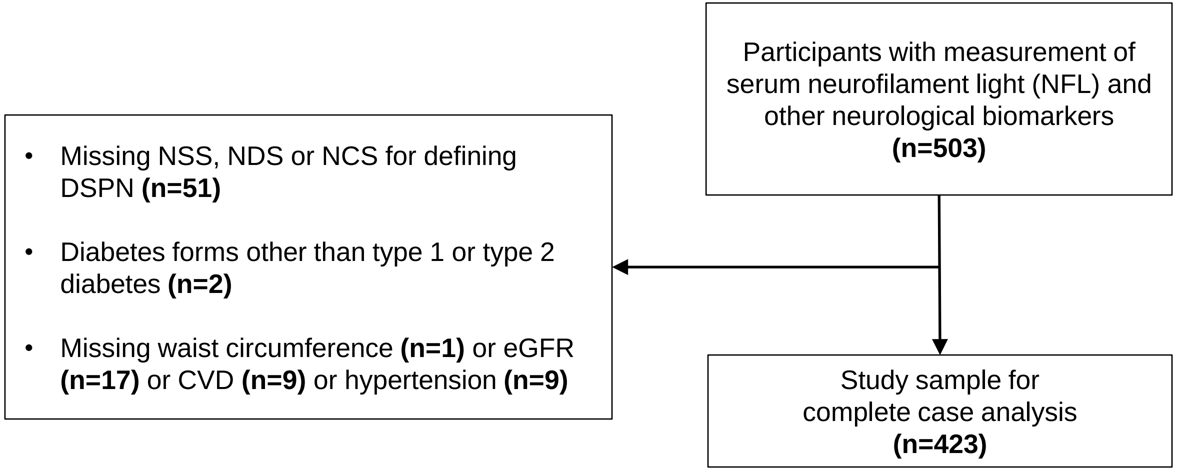

Note: some participants were excluded because of more than one exclusion criterion being applied

ESM Fig.2. Flowchart of biomarkers selection

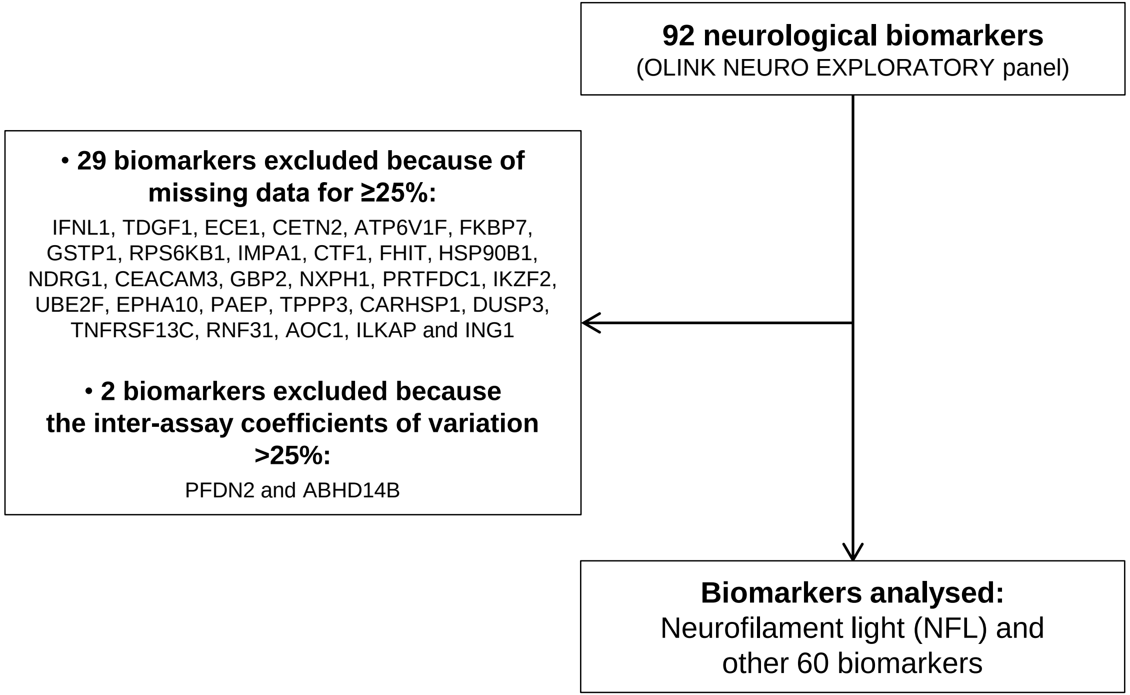

ESM Fig.3. Serum NFL levels by DSPN stages

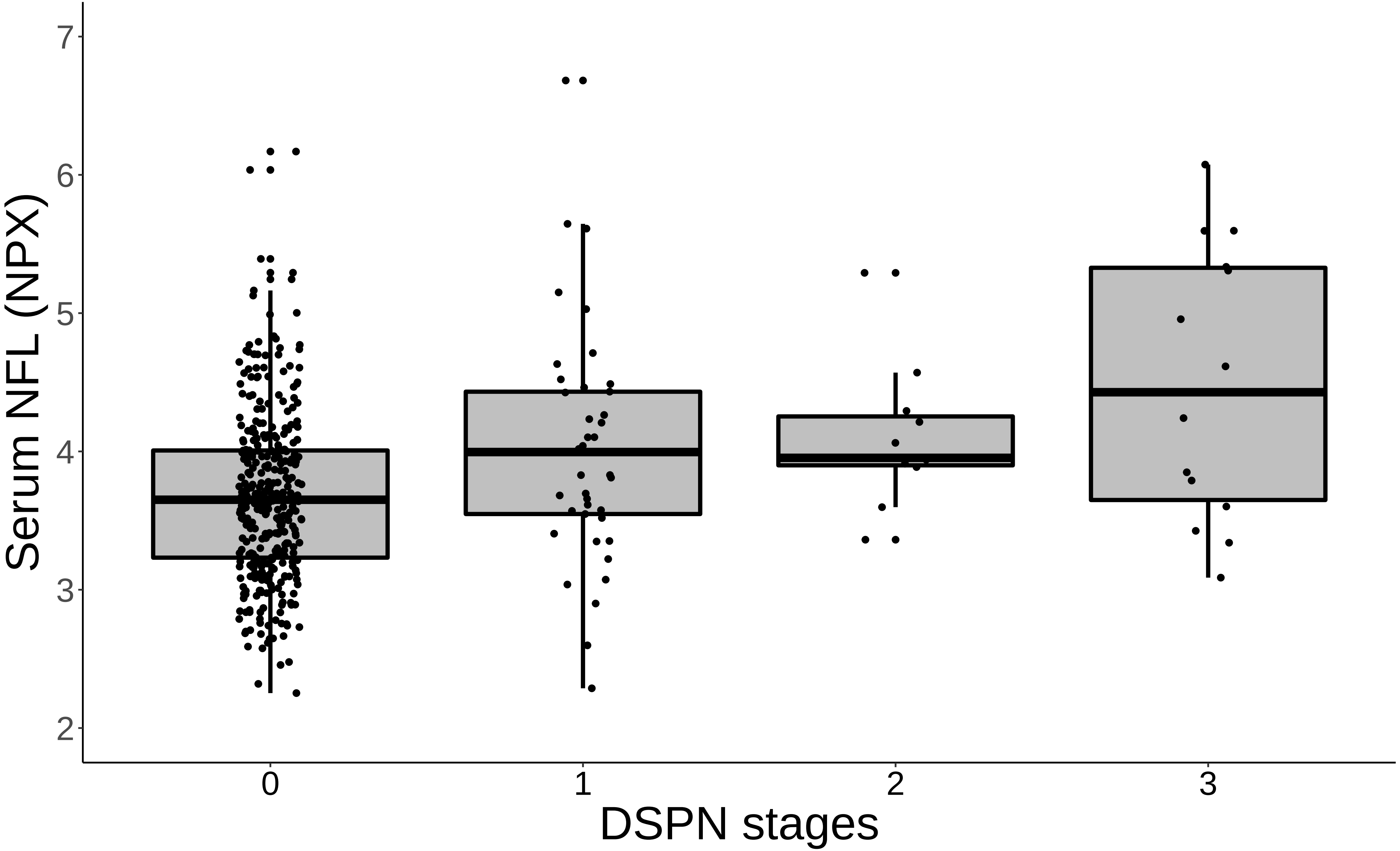

0= No DSPN, 1= Subclinical DSPN, 2= Confirmed asymptomatic DSPN, 3= Confirmed symptomatic DSPN
The boxplots with jittered data points show the distribution of serum NFL according to DSPN stage.
The line that divides the box into 2 parts represents the median of the data.
The ends of the box show the upper (Q3) and lower (Q1) quartiles.
The extreme line shows Q3+1.5xIQR to Q1−1.5xIQR

ESM Fig.4. Spearman correlation of serum NFL and age at diagnosis

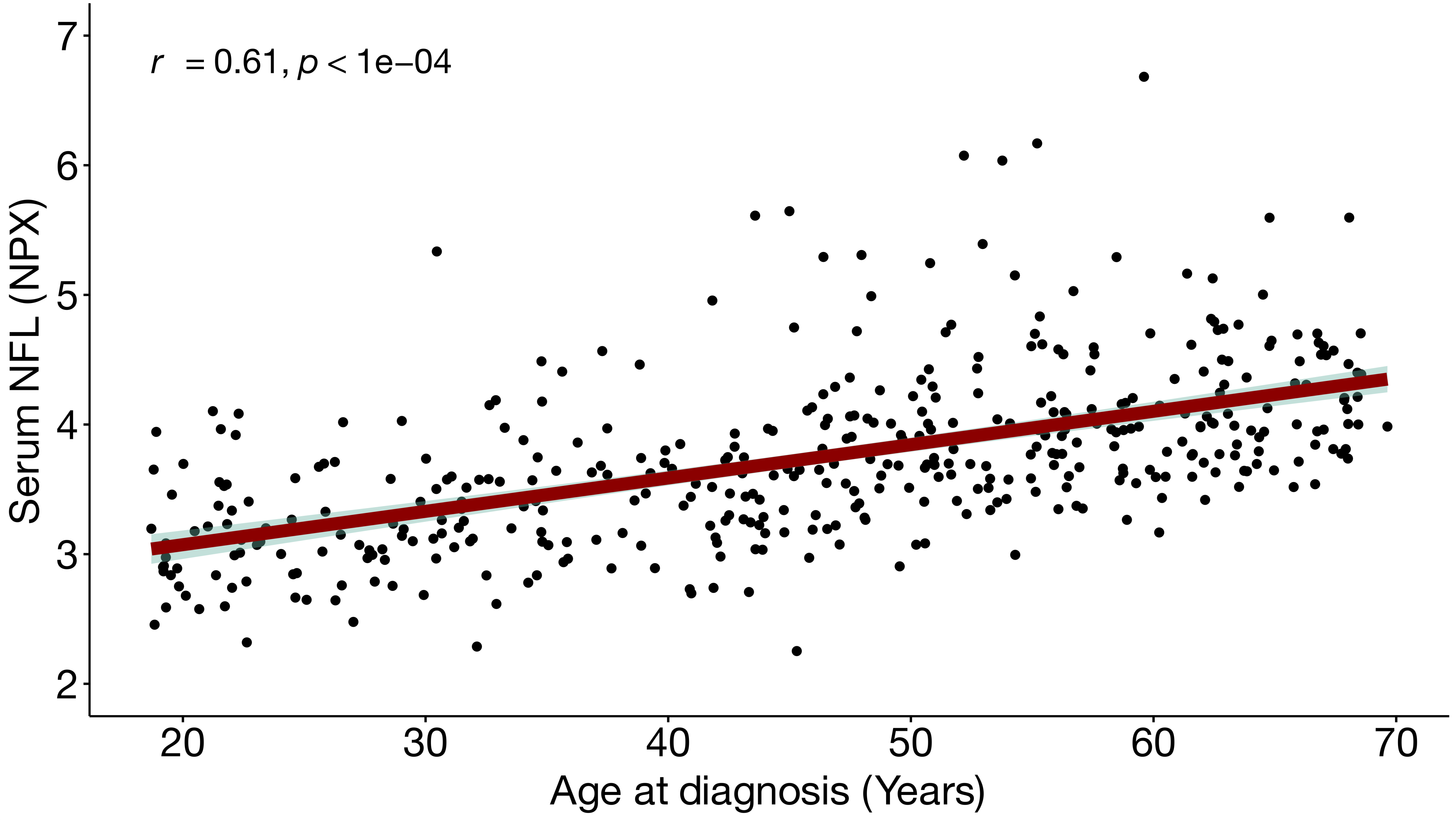

ESM Fig.5. ROC curve of serum NFL

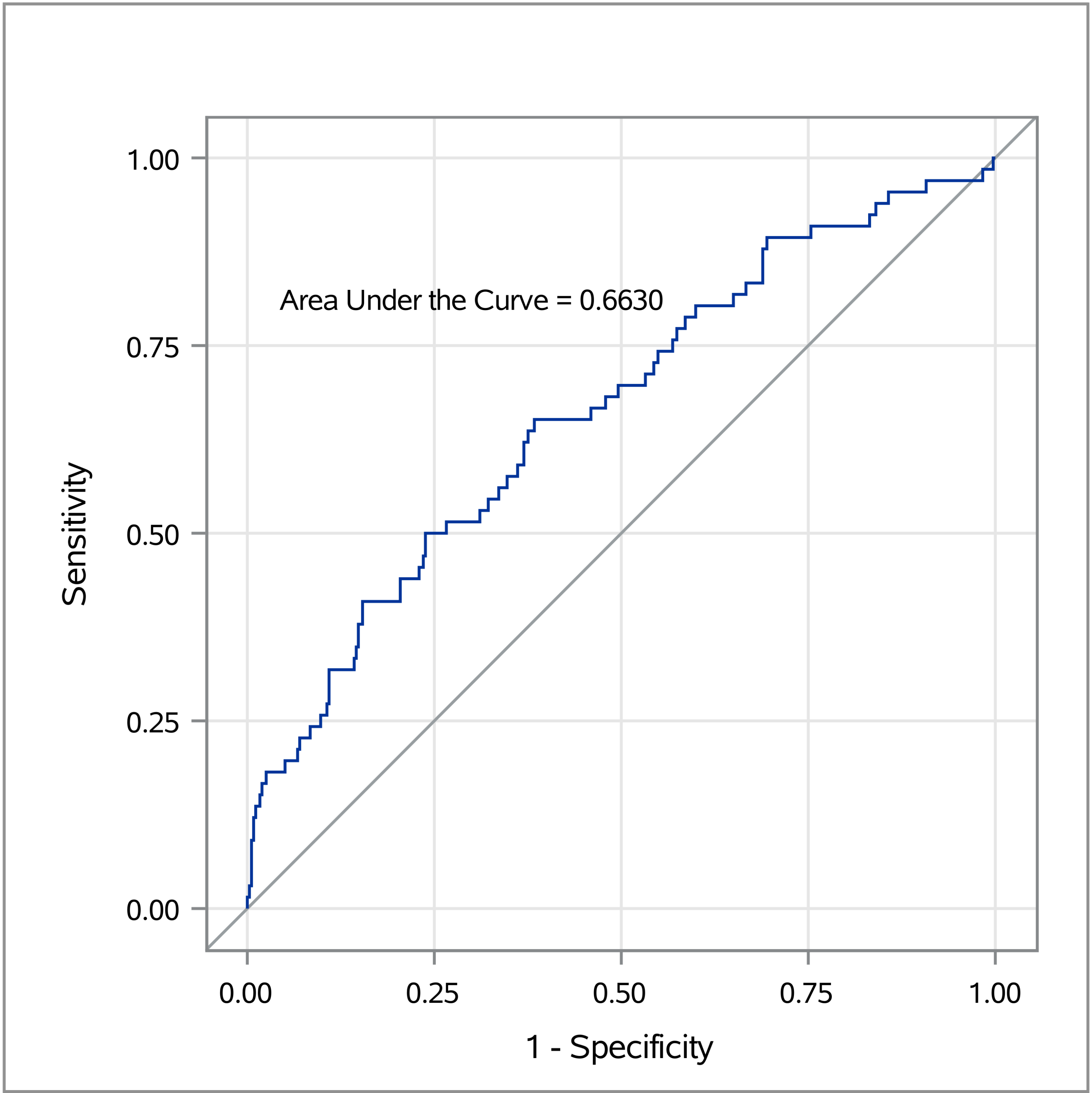

ESM Fig.6. Spearman correlations among all neurological biomarkers

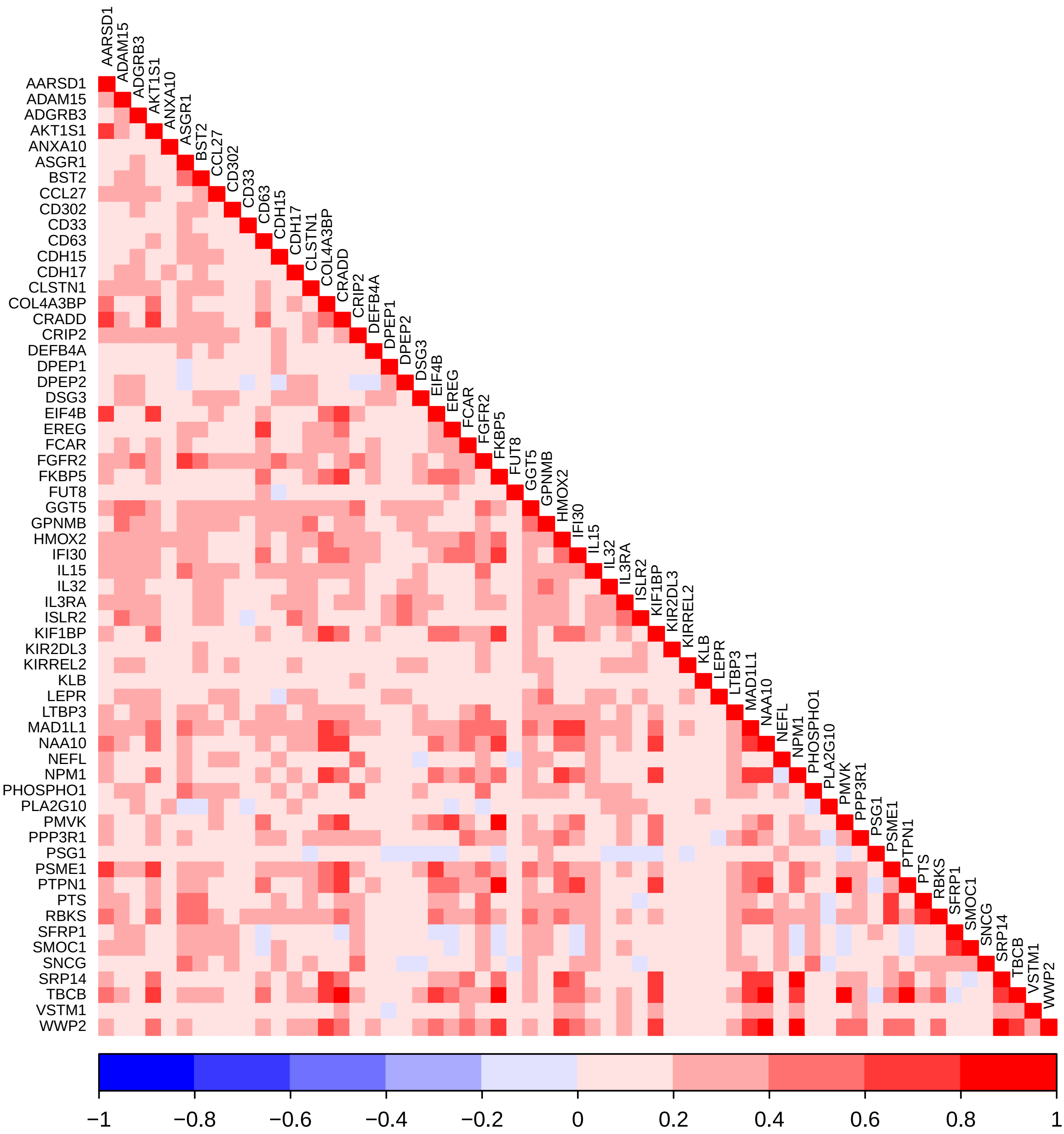

ESM Fig.7. Correlations of biomarkers with covariates

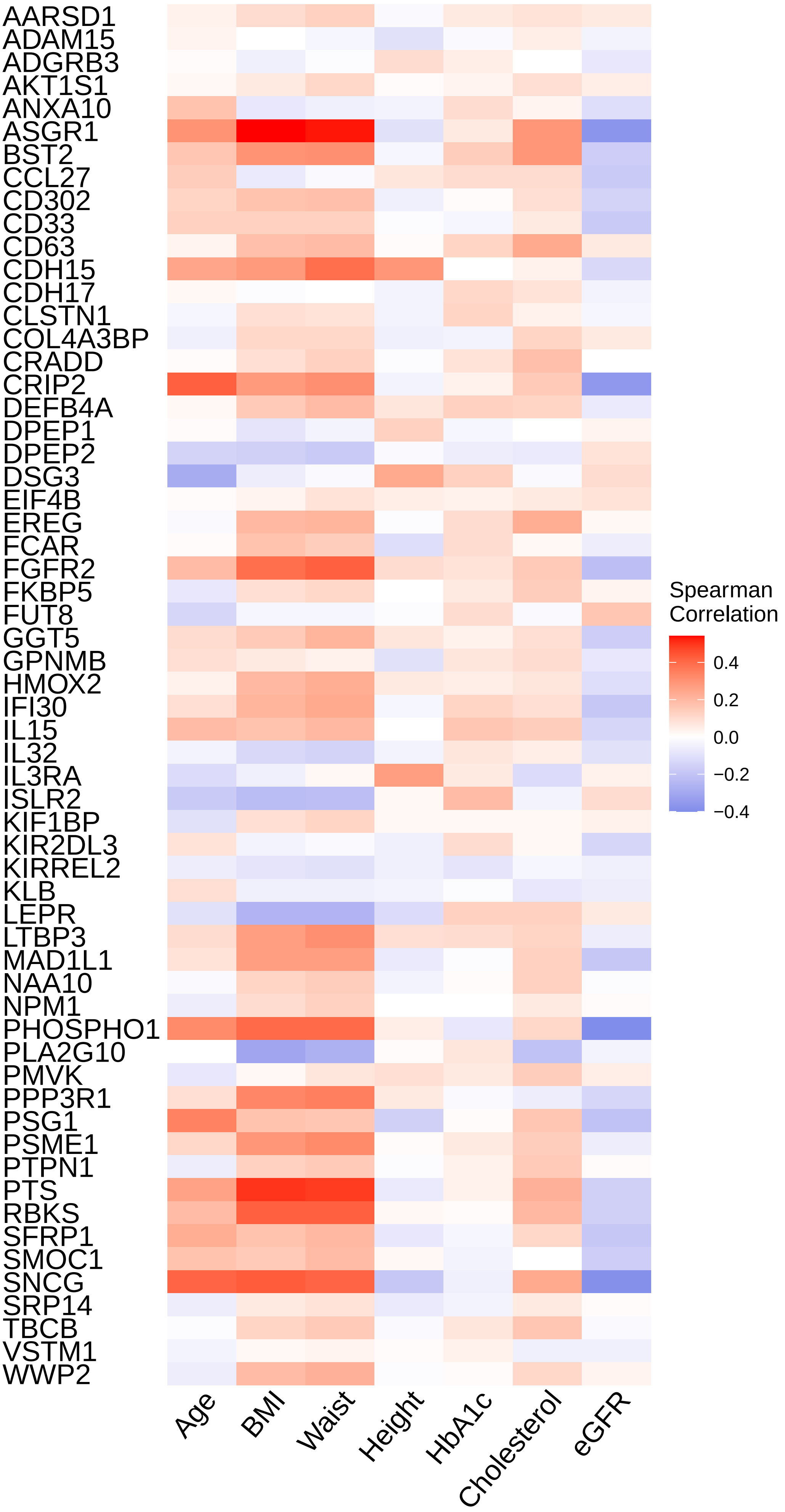

ESM Fig.8. Beta estimates for the associations of biomarkers (per 1−NPX increase) with nerve conduction velocities

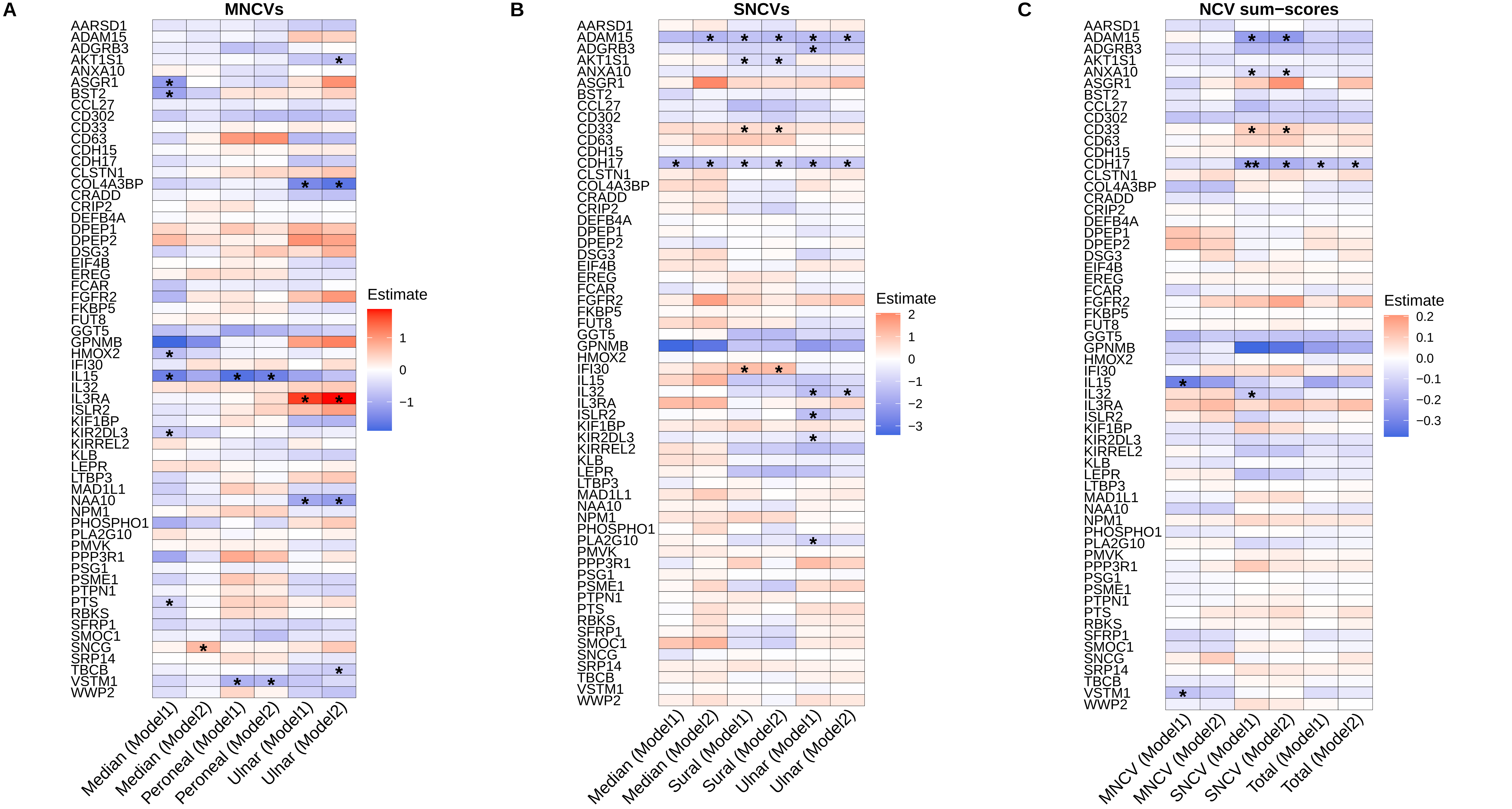

*p <0.05; **Bonferroni−corrected p<0.0008 indicates statistical significance, MNCV; motor nerve conduction velocity, SNCV; sensory nerve conduction velocity

ESM Fig.9. Beta estimates for the associations of biomarkers (per 1−SD increase) with nerve conduction velocities

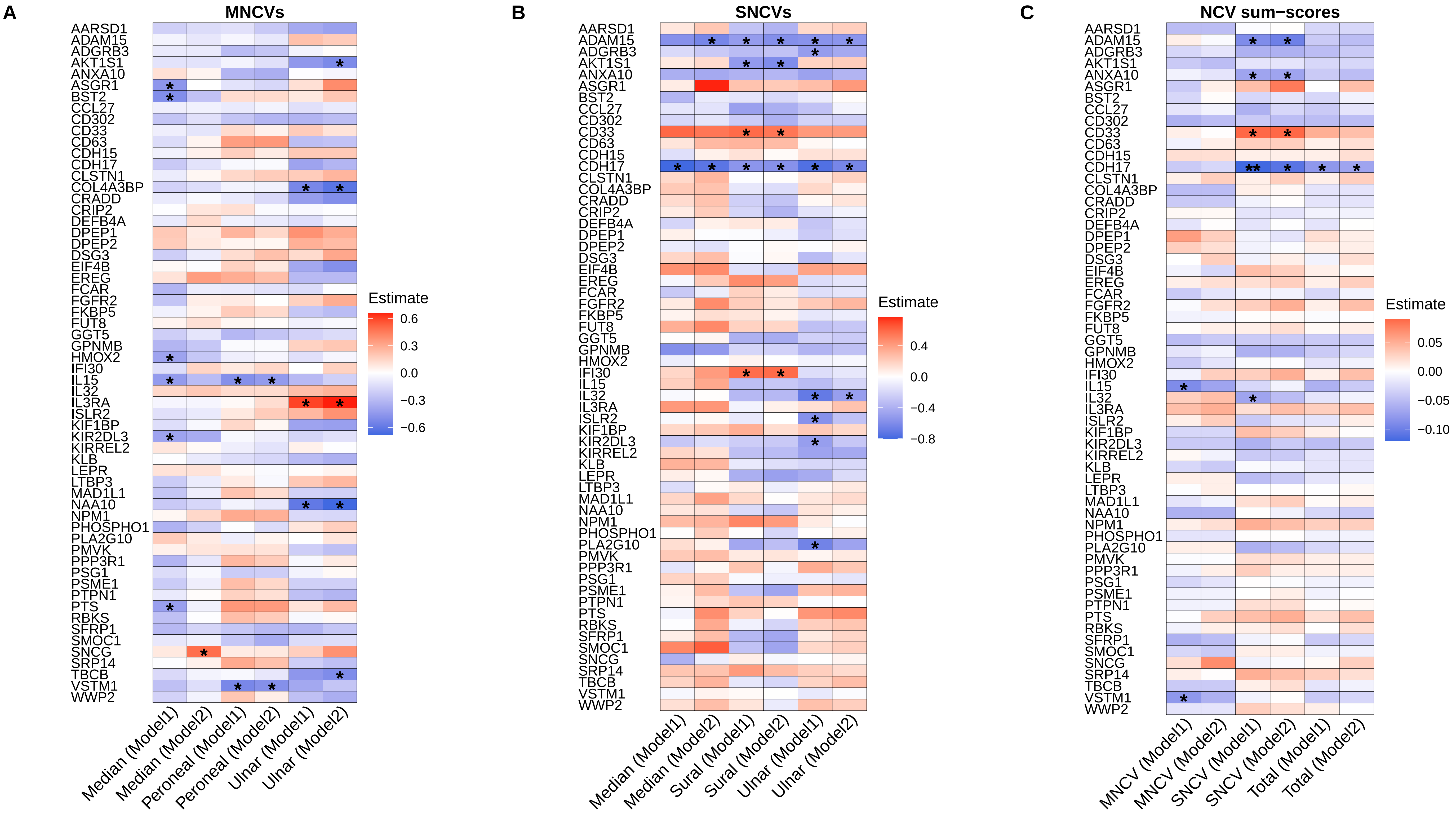

*p <0.05; **Bonferroni−corrected p<0.0008 indicates statistical significance, MNCV; motor nerve conduction velocity, SNCV; sensory nerve conduction velocity
